# Bimodal diel pattern in peatland ecosystem respiration rebuts uniform temperature response

**DOI:** 10.1038/s41467-020-18027-1

**Published:** 2020-08-26

**Authors:** Järvi Järveoja, Mats B. Nilsson, Patrick M. Crill, Matthias Peichl

**Affiliations:** 1 Department of Forest Ecology & Management, Swedish University of Agricultural Sciences, Umeå, Sweden; 2 Department of Geological Sciences, Stockholm University, Stockholm, Sweden; 3 Bolin Centre for Climate Research, Stockholm, Sweden

**Keywords:** Carbon cycle, Carbon cycle, Climate change

## Abstract

Accurate projections of climate change impacts on the vast carbon stores of northern peatlands require detailed knowledge of ecosystem respiration (ER) and its heterotrophic (Rh) and autotrophic (Ra) components. Currently, however, standard flux measurement techniques, i.e. eddy covariance and manual chambers, generate empirical ER data during only night- or daytime, respectively, which are extrapolated to the daily scale based on the paradigm that assumes a uniform diel temperature response. Here, using continuous autochamber measurements, we demonstrate a distinct bimodal pattern in diel peatland ER which contrasts the unimodal pattern inherent to the classical assumption. This feature results from divergent temperature dependencies of day- and nighttime ER due to varying contributions from Rh and Ra. We further find that disregarding these bimodal dynamics causes significant bias in ER estimates across multiple temporal scales. This calls for improved process-based understanding of ER to advance our ability to simulate peatland carbon cycle-climate feedbacks.

## Abstract (web summary)

Predicting the fate of carbon in peatlands relies on assumptions of behaviour in response to temperature. Here, the authors show that the temperature dependency of respiratory carbon losses shift strongly over day-night cycles, an overlooked facet causing bias in peatland carbon cycle simulations.

## Introduction

Northern peatlands have sequestered about 270–547 Gt of carbon (C) since the end of the last glacial period, which represents 20–30% of the organic C currently stored in soils worldwide^1–5^. Ecosystem respiration (ER) is the dominant pathway for C losses from peatlands to the atmosphere, and recently concern has been raised that the loss rate might increase under future climate change^6–9^. Understanding the dynamics and controls of peatland ER is therefore imperative for making accurate predictions of climate change impacts on the peatland C sink-source strength.

Conceptually, ER is the sum of two fundamentally different processes, i.e., microbial heterotrophic and plant autotrophic respiration (Rh and Ra, respectively). The magnitudes of each of these are the result of multiple soil biogeochemical and plant physiological processes^10–12^. This implies that the individual contributions of Rh and Ra to ER vary over space and time depending on biotic (e.g., plant species composition, biomass pools, phenology) and abiotic (e.g., air and soil temperature, solar radiation, water availability) conditions^10,13^. To accurately interpret the observed patterns in ER an in-depth mechanistic understanding of the spatio-temporal dynamics of Rh and Ra is essential.

Our current knowledge on the patterns and controls of diel peatland ER and its underlying components is, however, limited in no small part due to methodological constraints in the standard measurement techniques. Specifically, eddy covariance (EC) and manual dark chamber measurements provide only semi-continuous empirical ER data either at high frequency but limited to the nighttime (when the net CO_2_ exchange equals ER due to the absence of photosynthesis) or at coarse (e.g., weekly to biweekly) intervals and predominantly during only the daytime, respectively. Based on the well-established temperature dependency of respiration^14–16^, these periodic measurements are then extrapolated to the daily scale (and further to annual budgets) assuming a uniform diel temperature response^17,18^. However, given the additional underlying drivers and their diel variations, these simple temperature response functions are prone to fail in accurately simulating ER over the entire diel cycle^19,20^.

As an alternative, an automated dark chamber system provides direct ER estimates (in short-canopy ecosystems) at high temporal resolution (e.g., hourly) throughout the full diel cycle. While such systems have previously been used to measure diel respiration fluxes in forests^21–23^, grasslands^20,24^ and croplands^25^, similar studies are lacking in northern peatland ecosystems. As a result, our current knowledge of peatland ER dynamics and budgets relies heavily on the conceptual assumptions inherent to the models used for extrapolating semi-continuous diel data to daily and annual scales.

Here, we use an autochamber (AC) system installed across experimental plots^13^ to capture the complete diel cycles of ER and its underlying components (i.e., Rh and Ra) at an hourly resolution in a boreal minerogenous peatland over 3 years. To further investigate their separate abiotic and biotic controls, we explore the coherence of these diel fluxes with comprehensive environmental and vegetation data. Next, we compare our diel AC ER estimates with those obtained from extrapolating the nighttime AC data to the daytime to test a standard ER modeling approach^26^ widely used within the flux community (see Methods). We observe a distinct bimodal pattern in ER and a divergence in its response to temperature throughout the diel cycle, which emanates from the contrasting dynamics of Rh and Ra. These findings highlight the need for recognizing ER as a composite flux and for moving towards a detailed process-based understanding of its individual Rh and Ra components in northern peatlands.

## Results

### Bimodal diel pattern in peatland ER

**Fig. 1:**
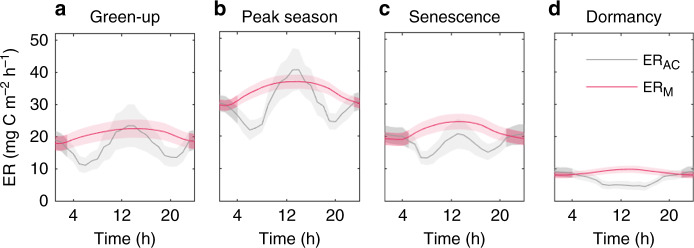
Diel patterns of measured versus extrapolated ecosystem respiration. Diel patterns of ecosystem respiration (ER) measured by autochambers (ER_AC_) and estimated by a standard flux modeling approach that extrapolated nighttime ER_AC_ data to the daytime (ER_M_) during the key phenophases of **a** green-up, **b** peak season, **c** senescence and **d** dormancy (spring and autumn) shown as a mean of the years 2015–2017 (see Supplementary Fig. 1 for ER_AC_ results from individual years). Shaded bands indicate ±1 standard error for a given hour resulting from the variation within each phenophase and across the 3 years. Darker shading shows the nighttime hours of the diel cycle. ER_M_ is modeled based on an exponential relationship between nighttime ER_AC_ and air temperature using the REddyProcWeb online gap-filling tool (Wutzler et al.^26^; see Methods for details).

Our continuous AC ER measurements revealed that ER peaked at both midday as well as around midnight from green-up to senescence (see ER_AC_ in Fig. 1a–c; Supplementary Fig. 1a–c). It is further noteworthy that the secondary nighttime maximum was of similar magnitude to the daytime one during the green-up and senescence phases. Meanwhile, maximum daytime ER was about 25% higher compared to the nighttime during the peak season. These observed bimodal diel ER patterns diverge from the unimodal ER dynamics obtained from extrapolating our nighttime AC ER data with the standard flux modeling approach^26^ (see ER_M_ in Fig. 1a–c). Similarly, the diel AC ER patterns contrast the unimodal diel ER estimates derived from adjacent (i.e., ~25 m) EC measurements which rely on the same modeling procedure (Supplementary Fig. 2a–c), i.e., extrapolating nighttime ER to the daytime assuming a uniform diel temperature response. Furthermore, while our AC data agree with the standard approach on a unimodal diel ER pattern during dormancy, they suggest a daytime minimum and nighttime maximum (Fig. 1d; Supplementary Fig. 1d) which is *de facto* reverse to the diel ER patterns of the model extrapolation (Fig. 1d) and EC-based estimates (Supplementary Fig. 2d).

**Fig. 2:**
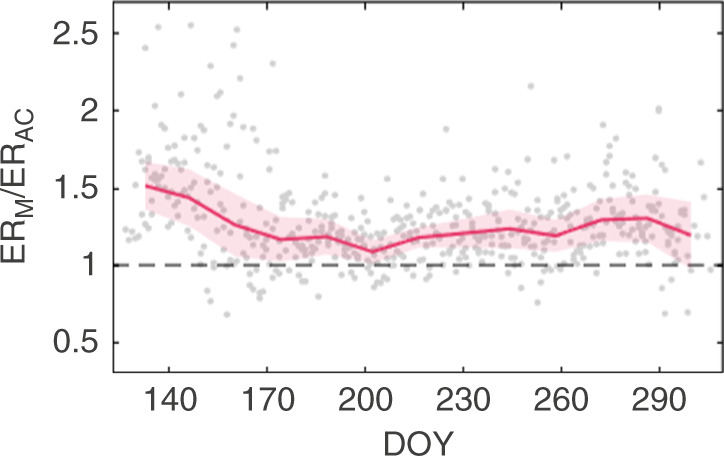
Daily bias in extrapolated ecosystem respiration estimates. Ratios of daily ecosystem respiration (ER) estimated by a standard flux modeling approach (ER_M_) and measured by autochambers (ER_AC_). Symbols indicate ratios of daily ER_M_ and ER_AC_ for the years 2015–2017; the red line represents the block-average (window size = 14 days) with shaded bands indicating ±1 standard error. Horizontal dashed line represents unity of the ratio. The 3-year means of the phenophase transition dates (DOY day of year) are as follows: 143 (spring dormancy → green-up), 187 (green-up → peak season), 218 (peak season → senescence), and 272 (senescence → autumn dormancy).

The divergence in the hourly measured and modeled (i.e., extrapolated) ER estimates was largest during the day-night transition periods (compare ER_AC_ and ER_M_ in Fig. 1). Over the course of the snow-free season, the ratio of the hourly modeled and measured ER estimates ranged from about 0.5 to >5 (Supplementary Fig. 3). We further find a consistent positive bias in daily ER estimates derived from the extrapolation of nighttime AC data (with up to two times higher values during the spring dormancy and green-up phases) compared to our measured daily ER (Fig. 2). Aggregating this daily bias resulted in an overestimation of cumulative ER over the snow-free season by 16, 17, and 22% in 2015, 2016, and 2017, respectively (see Supplementary Fig. 4). Conversely, we estimate that extrapolating daytime ER data from manual chamber measurements based on a uniform diel response to temperature would underestimate ER over the snow-free season on average by 17% (Supplementary Table 1).

**Fig. 3:**
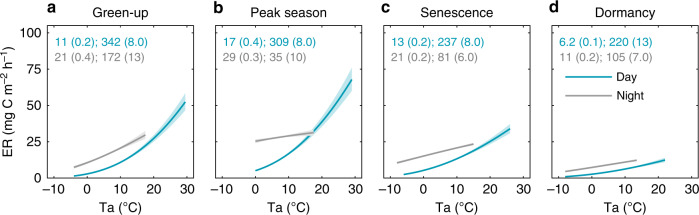
Diel divergence in the temperature response of ecosystem respiration. Exponential regression relationships between ecosystem respiration (ER) measured by autochambers and air temperature (Ta) for day- and nighttime (i.e., photosynthetic photon flux density ≥20 and <20 μmols m^−2^ s^−1^, respectively) during the key phenophases of **a** green-up, **b** peak season, **c** senescence and **d** dormancy (spring and autumn) shown as a mean of the years 2015–2017 (see Supplementary Fig. 5 for results from individual years). Values shown in the panels (blue and gray for day- and nighttime periods, respectively) represent model parameters *R*_10_ and *E*_0_ with standard errors in brackets from the Lloyd & Taylor^14^ respiration model (see equation in Methods). Solid lines indicate the exponential fit and shaded bands indicate the 95% confidence intervals.

In contrast to the uniform diel relationship between ER and air temperature (Ta) assumed by the standard approach to extrapolate semi-continuous ER measurements, the response of our measured AC ER to changes in Ta varied between day- and nighttime (Fig. 3). Specifically, the parameter representing base respiration rates at 10 °C (*R*_10_) in the Lloyd & Taylor respiration model^14^ (see equation in Methods) was consistently about two times higher during nighttime compared to daytime in each phenophase. Meanwhile, the model parameter describing the sensitivity of respiration to a change in temperature (*E*_0_) was always significantly higher during the daytime. The magnitudes of the deviations between day- and nighttime parameters, however, varied throughout the different phenophases (Fig. 3) as well as among the individual years (Supplementary Fig. 5).

### Diel patterns and controls of Rh and Ra

To help explain the observed diel patterns and temperature dependency of ER, we investigated the underlying Rh and Ra fluxes as well as their controls using an in situ flux partitioning approach with natural and experimental trenching/vegetation removal plots.

**Fig. 4:**
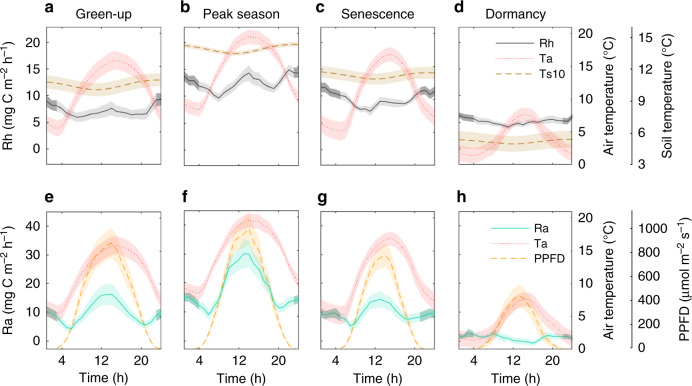
Diel patterns of heterotrophic and autotrophic respiration. Diel patterns of **a**–**d** heterotrophic respiration (Rh) and **e**–**h** autotrophic respiration (Ra) and their main abiotic controls including air temperature (Ta), soil temperature at 10 cm depth (Ts10) and photosynthetic photon flux density (PPFD) during the phenophases of green-up, peak season, senescence, and dormancy (spring and autumn) shown as the mean over the years 2015–2016 (see Supplementary Fig. 6 for results from individual years; no Rh and Ra data were available in 2017). Shaded bands indicate ±1 standard error for a given hour resulting from the variation within each phenophase and across the measurement years. Darker shading in Rh and Ra shows the nighttime hours of the diel cycle.

**Fig. 5:**
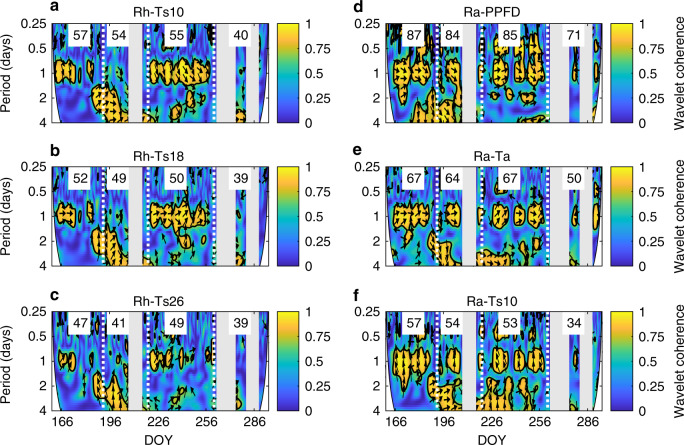
Abiotic controls of heterotrophic and autotrophic respiration. Wavelet coherence (yellow = strong and blue = absent) between hourly fluxes of **a**–**c** heterotrophic respiration (Rh) and **d**–**f** autotrophic respiration (Ra) and their abiotic controls (i.e., soil temperature at 10, 18, and 26 cm depth (Ts10, Ts18, and Ts26), photosynthetic photon flux density (PPFD) and air temperature (Ta)) in 2015 (see similar results for 2016 in Supplementary Fig. 7; no Rh and Ra data were available in 2017). Arrows indicate lag: right = no lag, up or down = quarter diel cycle (i.e., 6 h) lag of the flux behind or ahead of environmental variable, respectively, left = antiphase lag (i.e., 12 h). Gray vertical bands indicate extended periods with missing hourly Rh and Ra data. Vertical dotted lines indicate transitions between the phenophases (green-up → peak season → senescence → autumn dormancy). Numbers in white boxes represent the Welch’s cross power spectral density values between the hourly flux and environmental variables at the 1-day period (see also Supplementary Fig. 9). DOY stands for day of year.

We found that Rh fluxes were generally lower during the daytime and reached a maximum during the midnight hours in all growth phases (Fig. 4a–c; Supplementary Fig. 6a–c). During the peak season, an additional increase in Rh occurred around noon (Fig. 4b; Supplementary Fig. 6b). In contrast, during dormancy Rh followed a weak diel pattern with somewhat higher fluxes occurring during the night (Fig. 4d; Supplementary Fig. 6d). A wavelet coherence analysis revealed that the observed diel patterns of Rh followed most closely those of soil temperature (Ts) (Fig. 5a–c; Supplementary Fig. 7a–c) while being anticyclical to those of Ta (Supplementary Fig. 8a, b) and only weakly coherent with those of water table level (WTL) (Supplementary Fig. 8c, d) throughout the various phenophases. However, the intermittent daytime increase in Rh observed during the peak season phase coincided with the daytime maxima in Ta (Fig. 4b). Across the measured levels, Ts at the 18 cm depth was most closely in sync with Rh while forward and backward lags of several hours were observed for temperature at higher (i.e., 10 cm) and lower (i.e., 26 cm) depths, respectively. Meanwhile, Welch’s cross power spectral density values were highest for Ts at the 10 cm depth in each phenophase (Fig. 5a–c; Supplementary Figs. 7a–c, 9a–d). Overall, the correlations with temperature at single given depths remained, however, relatively weak. When further separating the phenophases into dry and wet conditions, we found that during the peak season both day- and nighttime Rh fluxes were higher (with similar relative increases) under dry conditions (Supplementary Fig. 10b). In comparison, WTL effects on the diel Rh patterns were limited during the other phenophases (Supplementary Fig. 10a, c, d).

The diel variations of plant-associated respiration, i.e., Ra, featured a similar though somewhat less pronounced bimodal pattern as ER. Specifically, maximum Ra occurred around noon during the green-up, peak season, and senescence phases (Fig. 4e–g; Supplementary Fig. 6a–c) exceeding that of Rh by about twofold. An additional relatively smaller peak was evident during the midnight hours reaching similar magnitudes as the concurrent Rh fluxes. As to be expected, no clear diel pattern occurred in Ra during dormancy (Fig. 4h). Results from the wavelet coherence analysis suggest that from green-up to senescence the diel patterns of Ra during daytime were regulated primarily by the temporal dynamics of the photosynthetic photon flux density (PPFD) and Ta (Fig. 5d–f; Supplementary Fig. 7d–f). In particular, the timing for the onset of the morning increase in Ra followed shortly that of Ta and PPFD while the onset of the afternoon decrease in Ra coincided with that of PPFD which showed an earlier and steeper decline compared to Ta (Fig. 4e–h). An assessment of the Welch’s cross power spectral density confirmed that PPFD was overall a stronger control than Ta of the diel Ra pattern in each of the phenophases (Fig. 5d–f; Supplementary Fig. 9e–h). We further found that dry conditions in the peak season and senescence phases coincided with enhanced Ra, with relatively larger increases during day- compared to nighttime (Supplementary Fig. 10b, c). Averaged over the snow-free season, the spatial variation in the diel amplitude of Ra exceeded that of Rh with daytime maximum Ra generally corresponding to differences in total green biomass across the four replicate chamber groups (Supplementary Fig. 11).

**Fig. 6:**
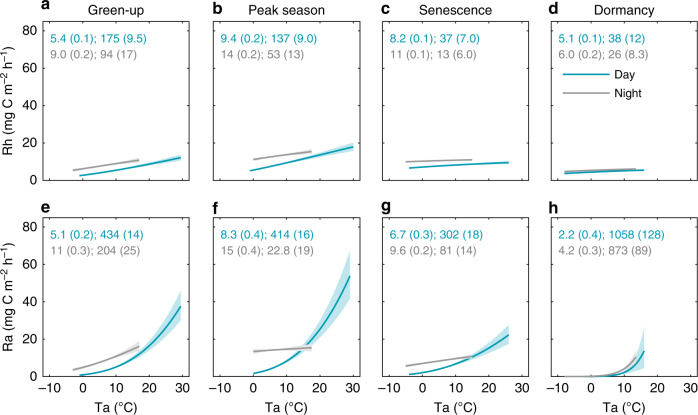
Diel temperature responses of heterotrophic and autotrophic respiration. Exponential regression relationships between **a**–**d** heterotrophic respiration (Rh) and **e**–**h** autotrophic respiration (Ra) and air temperature (Ta) for day- and nighttime (i.e., photosynthetic photon flux density ≥ 20 and <20 μmols m^−2^ s^−1^, respectively) during the key phenophases of green-up, peak season, senescence, and dormancy (spring and autumn) shown as a mean of the years 2015–2016. Values shown in the panels (blue and gray for day- and nighttime periods, respectively) represent model parameters *R*_10_ and *E*_0_ with standard errors in brackets from the Lloyd & Taylor^14^ respiration model (see equation in Methods). Solid lines indicate the exponential fit and shaded bands indicate the 95% confidence intervals.

Our flux partitioning approach further revealed considerable differences in the temperature response between Rh and Ra as well as across diel and seasonal scales (Fig. 6), which altogether explain the observed variability in the temperature dependency of ER (Fig. 3). It is noteworthy that the day-nighttime hysteresis observed in the response of Rh to Ta (Fig. 6a–d) is largely reduced (except during senescence) when using Ts as the temperature driver (see Supplementary Fig. 12a–h). Further modifications of the temperature relationships were noted particularly for Ra and to a lesser extent for Rh during dry and wet periods (i.e., periods with a WTL below or above its respective phenophase mean) as a function of WTL fluctuations (for details see Supplementary Fig. 12).

## Discussion

Our empirical evidence for bimodal diel ER patterns strongly contrasts with the established narrative of diel ER having a unimodal pattern emerging from the assumption of a uniform temperature response^17,26,27^. Our results further show that not considering the bimodal response may result in a significant bias in peatland ER estimates from hourly to annual scales. The implications from these findings are potentially far-reaching since this classical assumption is the foundation based on which many field studies extrapolate semi-continuous empirical ER data from EC^17,26^ and manual dark chamber^28,29^ measurements to the daily scale.

In particular, the manifold bias observed at hourly and daily scales significantly limits our process-based understanding of the ER response to external perturbations occurring at these shorter timescales (e.g., rainfall events, heat waves, and other weather patterns). The accumulated bias in ER over the snow-free season (ranging from 15 to 17 g C m^−2^ during the 3 measurement years) is also considerable since it represents about one quarter of the long-term mean annual net CO_2_ sink (NEE; 58 ± 21 g C m^−2^)^30^ and about two-thirds of the annual net ecosystem carbon balance (NECB; 23.5 g C m^−2^)^31^. In addition, we estimated that using the manual chamber approach in which daytime relationships between ER and Ta are extrapolated to the nighttime would overestimate the cumulative net CO_2_ uptake during the snow-free season by 19% at our peatland site (see Supplementary Table 1 for more details). In comparison, such bias in ER does not affect the direct net CO_2_ exchange estimates by the EC technique. However, many process-based peatland models^32–34^ and remote sensing proxies^35–37^ are developed (i.e., calibrated and validated) with the support of EC-derived ER estimates. Consequently, any bias in these observational data may not only hamper our understanding of processes at (sub-)daily timescales but also directly transfer into modeling estimates of regional and global peatland C budgets^38,39^.

We show that the observed bias emanates from the failed assumption of a uniform relationship between ER and temperature^17,29^ for both day- and nighttime conditions. Supported by previous studies reporting a diel hysteresis between respiration and temperature in forest^21,22^ and grassland^20,24^ systems, our study demonstrates a divergence in both parameters of the exponential model describing ER response to air temperature between night- and daytime. In addition, we show that the magnitude of this divergence varies throughout the different phenophases. This highlights that despite the intrinsic temperature dependency of individual plant and microbial respiration processes, the whole system response is determined by their integrated sensitivity to the different temperature regimes aboveground and at various depths belowground. Combined with the effects from additional abiotic (e.g., radiation and water availability) and other biotic (e.g., phenology) drivers, this will in sum create diel ER patterns which contrast those expected from a uniform diel response to a single temperature parameter. Thus, our findings indicate a need for redefining our conceptual assumptions regarding diel ER dynamics in empirical and process-based modeling studies of the northern peatland C cycle.

Our experimental flux partitioning approach further revealed that the bimodal ER pattern resulted from the separate diel dynamics of Ra and Rh that reached their respective maxima at midday and midnight. Given the well-established temperature dependency of respiration^14–16^, diel Rh minima and maxima occurring during warmer day- and colder nighttime conditions, respectively, might at first appear counterintuitive. However, this phenomenon is resolved by the observed dependency of Rh to soil rather than air temperature with the former displaying a temporal shift in the diel maxima and minima with depth toward midnight and noon hours, respectively. Similarly, increases in soil respiration during the evening hours have also been previously noted in forest and cropland ecosystems^21,23,25^. It is noteworthy that the identified specific peat depth with highest coherence between its diel temperature dynamics and bulk Rh flux (i.e., the surface efflux) might not necessarily be the location of highest decomposition rates. Instead, it might merely represent the layer whose diel temperature pattern coincides best with the mean of the various diel decomposition dynamics occurring in the profile layers above and below. The observation that the most coherent peat depth was located below the mean WTL further demonstrates that relatively lower decomposition rates in the inundated anaerobic peat layers still contribute considerably to total Rh as they integrate over substantial depths (i.e., >1 m)^40–45^. The combined contribution from the various layers to the bulk Rh flux measured at the soil surface also explains its relatively weak correlations to temperature at a single peat depth. Thus, our results highlight that the diel variation in the bulk Rh flux is regulated by a complex integral of depth-specific temperature dynamics with further modifications from water availability and substrate characteristics across the entire peat profile^42,46,47^. Compared to other terrestrial ecosystems (in particular tall canopy systems such as forests), peatlands are characterized by a relatively larger soil organic matter pool (i.e., up to several meters of peat) that sustains Rh and a smaller vegetation biomass pool, which limits the magnitudes of Ra. Thus, an enhanced contribution from diel Rh dynamics (relative to other ecosystem types) may facilitate a bimodal diel ER pattern as a unique feature for northern peatland ecosystems.

Meanwhile, the observed bimodal diel pattern in Ra may be explained by the individual dynamics in above- and belowground plant (i.e., rhizosphere) respiration processes in response to contrasting drivers. In particular, our results show that the pattern and magnitude of daytime Ra were strongly regulated by incoming solar radiation, air temperature, and water availability which are known drivers of vascular plant and moss photosynthesis^48^. In contrast, the secondary increase in Ra during nighttime might be due to elevated peat temperature and subsequently root respiration in combination with a lag in belowground allocation of recent photosynthates^21,49,50^ resulting in enhanced belowground Ra. However, the length of such lags in non-treed ecosystems has been reported to be highly variable ranging from hours to days depending on vegetation and soil hydrological characteristics^25,50^. Thus, the diel Ra (and subsequently ER) patterns might strongly depend on the site-specific timing of this allocation lag. The observed variations in maximum day- and nighttime Ra throughout the different phenophases (Fig. 4e–h) and in dependence of plant biomass (Supplementary Fig. 11b) further illustrate the important role of plant phenology (i.e., seasonal changes in biomass pools, photosynthetic capacity, and partitioning of above- to belowground production) in regulating Ra at seasonal and spatial scales. Overall, its greater variability and sensitivity to radiation (i.e., cloudiness), vegetation characteristics (Supplementary Fig. 11), and WTL (Supplementary Figs. 10 and 12) imply that Ra rather than Rh is the dominating component regulating the variations in the diel patterns and temperature response of ER.

Altogether, our results highlight that shifts in abiotic and biotic drivers due to climatic changes (including temperature, water balance, and cloudiness-dependent radiation regimes) might have contrasting effects on peatland ER depending on the individual responses of Rh and Ra. However, more evidence is needed to identify how general these findings on the diel respiration fluxes are across peatland ecosystems, the biophysical factors that regulate it, and the implications for estimating ER from sub-daily to annual scales. This further calls for incorporating automated chamber measurements of diel ER and its component fluxes as a new standard in international C flux monitoring networks (e.g., ICOS, NEON).

## Methods

### Study site

The study was carried out at Degerö Stormyr (64°11′N; 19°33′E), which is an oligotrophic minerogenous mire (i.e., a nutrient-poor fen) in northern Sweden (Västerbotten County). The site is situated within the boreal region with a climate that can be classified as cold temperate humid. Previous studies at the site suggest a long-term (2001–2012) mean annual air temperature of 2.3 °C and total annual precipitation of 666 mm^30^. The snow-free season lasts for about 6 months from around early May to late October. The WTL relative to the mire surface within our measurement area ranges between ~0 and −15 cm with a snow-free season mean of −4.7 cm^13^. The vegetation cover at the site consists of both *Sphagnum* spp. mosses as well as vascular plants. Specifically, the dominant species in the moss layer are *S. majus* Russ. C. Jens., *S. lindbergii* Schimp. and *S. balticum* Russ. C. Jens. The most typical vascular plant species include the sedges *Eriophorum vaginatum* L., *Trichophorum cespitosum* L. Hartm. and *Scheuzeria palustris* L. as well as the shrubs *Vaccinium oxycoccos* L. and *Andromeda polifolia* L^31,51^.

### Automated chamber system and experimental design

A custom-made automated chamber system (based on the design by Goulden & Crill^52^; Bubier et al.^53^) was installed at the site during spring 2014 in close vicinity of the EC flux tower^30^. A detailed description of the experimental design and the technical specifications of the chamber system are presented by Järveoja et al.^13^. In brief, the set-up includes four replicate groups, each consisting of three adjacently placed chambers (45 × 45 × 15 cm): one transparent chamber for measuring the net ecosystem exchange of carbon dioxide (NEE) and two dark chambers for measuring ER and heterotrophic respiration (Rh). In this study, we present the ER data collected during the years 2015–2017. Due to frequent flooding of the vegetation removal (i.e., Rh) plots during the wet year 2017, detailed flux partitioning data for autotrophic respiration (Ra) and Rh was available only in 2015 and 2016.

The ER chambers are located within the natural (i.e., undisturbed) lawn microforms of the peatland. Meanwhile, the Rh chambers are established on experimental plots (1 m^2^) where all photosynthetic biomass (i.e., vascular plants and the upper ~5 cm of the moss layer) was removed in autumn 2013. These plots were covered with artificial air and water permeable grass mats to maintain a similar surface albedo. In addition, the plot edges were trenched (to ~40 cm depth) and new sporadically emerging shoots were clipped. A comparison of Rh fluxes during 2014–2016 suggests that the initial clipping in 2013 had no noticeable effect on Rh in the subsequent years. It is, however, important to note that such trenching/vegetation removal treatments can generally not separate Ra from the fraction of Rh that is associated with the microbial metabolism of fresh root exudates (i.e., originating from the belowground allocation of plant photosynthates)^54^. Our flux partitioning approach thus somewhat underestimates the contribution from microbial respiration of these recently assimilated labile C sources while subsequently overestimating the relative contribution of Ra to ER. We expect, however, that this shortcoming has a limited impact on our results since this method-induced bias remains fairly constant and thus is unlikely to alter the diel patterns of Ra and Rh.

Each chamber structure consists of a rectangular aluminum frame and a moving chamber. All chambers are made of transparent Lexan polycarbonate, however, the ER and Rh chambers were made dark using reflective aluminum tape. The frame includes a 10 cm deep skirt mounted at the bottom and a water groove on the top to ensure an airtight seal during the sampling. The chambers are connected in a closed loop to a cavity ringdown greenhouse gas analyzer (Model GGA-24EP, Los Gatos Research Inc., San Jose, CA, USA).

One chamber measurement cycle consists of three consecutive steps: (1) 1 min flushing of the sample tubing with ambient air while the chamber is still open, (2) followed by 3 min of concentration measurements during which the chamber is closed, and (3) concluded by another 1 min flushing of the sampling tubing once the chamber is open again. This results in a total measurement cycle of 5 min per chamber and consequently one measurement per hour from each of the 12 chambers. The measurement sequence is kept constant, i.e., cycling from group 1 to group 4, with one NEE, ER, and Rh measurement conducted within each group.

In each chamber, air temperature (Ta) is continuously monitored 10 cm above the mire surface with thermocouple wires (Type K, PFA insulated, 0.25 mm diameter; Omega Engineering Inc., Norwalk, CT, USA). In addition, PPFD is quantified in all clear chambers using quantum sensors (Model SQ-110, Apogee Instruments Inc., Logan, UT, USA). Soil temperature (Ts) is measured at 2 and 10 cm depths within each chamber with thermistor probes (Model TO3R, TOJO Skogsteknik, Bygdeå, Sweden). Additional data for Ta (measured at 2 m height above the surface) and Ts at 2, 10, 18, 26, 34, and 42 cm depths are available from a nearby (~50 m) climate station^30^. Water table level (WTL) is continuously monitored at each chamber group with a submersible pressure transducer (Model CS451, Campbell Scientific, Logan, UT, USA).

### Data processing, flux calculation, and quality control

The data processing, flux calculation, and quality control procedures have been described in detail by Järveoja et al.^13^. In brief, flux rates were computed from the linear change in the headspace carbon dioxide concentration over time (after discarding the first 20 s following chamber closure) corrected for air density using the ideal gas law. Hourly means of ER, Ra, and Rh were calculated from the available replicate measurements within each hour (i.e., between 1 and 4). Uncertainty estimates for each hourly treatment mean flux were computed based on the 95% confidence intervals considering the number of chamber fluxes available in each hour. Hourly mean estimates of Ra were derived from the independent measurements of the ER and Rh fluxes as Ra = ER − Rh. All fluxes are expressed following the atmospheric sign convention in which positive fluxes represent emission to the atmosphere.

A number of quality control procedures were applied to detect and discard poor quality flux data. Specifically, this included: (1) filtering out fluxes with an RMSE > 0.5 and *R*^2^ < 0.95 (*P* < 0.001), (2) removing sporadic negative (i.e., apparent uptake) fluxes in ER and Rh, (3) eliminating Rh fluxes when the WTL was higher than −5 cm from the mire surface (since the experimental plots were flooded in these conditions due to a lower surface level following the vegetation removal) and (4) filtering and correcting fluxes measured during calm nights. The last step is necessary to account for the apparent increase in nighttime flux estimates, which results from the artifact that may occur when the closing chamber disturbs the steep concentration gradient above the mire surface common for stable atmospheric conditions (see e.g., Lai et al.^55^; Brændholt et al.^56^). For this purpose we corrected our nighttime flux estimates with the approach developed by Järveoja et al.^13^. To ensure that our nighttime correction approach removed the potential bias from CO_2_ accumulation during calm stable nights, we created an alternative dataset in which we removed all nighttime flux data with ambient CO_2_ concentrations ≥ 415 ppm. A comparison of the diel patterns obtained from the original and filtered datasets suggests only negligible differences (likely resulting from the fact that the two datasets comprise different periods with varying environmental conditions) (Supplementary Fig. 13) demonstrating that our nighttime correction approach effectively eliminated the apparent bias. We further note that methane fluxes measured with the same system do not show such nighttime increase when corrected with the same approach (Supplementary Fig. 14). Altogether, this suggests that nighttime patterns in respiration fluxes observed in our final quality controlled data are due to biological processes rather than the result of a systematic artifact due to accumulation of CO_2_ during calm nights.

The relationships of ER, Rh, and Ra to temperature were estimated using the exponential model Eq. (1) by Lloyd & Taylor^14^:

1 $$R \,=\, R_{10}{\mathrm{exp}}^{E_0\left( {\frac{1}{{56.02}} - \frac{1}{{T - 227.13}}} \right)},$$

where *R* is ER, Rh, or Ra, *T* is temperature (i.e., Ta or Ts), *R*_10_ represents base respiration at 10 °C and *E*_0_ represents the activation energy parameter, i.e., the sensitivity of respiration fluxes to changes in temperature.

### Alternative ER estimates from a standard modeling approach

For comparing the diel ER patterns from our continuous AC measurements to those commonly obtained from extrapolation of discontinuous data, we derived an alternative ER dataset using the REddyProcWeb online tool^26^. This tool is the standard modeling approach within the EC flux community used to extrapolate EC-based nighttime measurements of NEE (i.e., ER) to the daytime based on exponential relationships with (air or soil) temperature^17,26^. To achieve this, we provided the automated chamber ER data along with key micrometeorological variables (i.e., global radiation, air temperature, relative humidity, vapor pressure deficit, and friction velocity) obtained from the nearby (~25 m) climate station as input for the online tool algorithm which subsequently simulated diel ER. We further investigated the diel patterns of EC-based ER fluxes. For this purpose, we obtained readily available half-hourly ER estimates derived from EC measurements at the adjacent ICOS-Degerö station (www.icos-sweden.se/station_degero.html) using the REddyProcWeb online tool.

### Vegetation phenology

To track the seasonal vegetation biomass development with high temporal resolution, a greenness index (i.e., the green chromatic coordinate; gcc) was derived from hourly images collected through digital repeat photography as described in detail by Peichl et al.^57^ and Järveoja et al.^13^. We further used gcc to determine the distinct phenophases of (1) green-up, (2) peak season, (3) senescence, and (4) dormancy (i.e., the snow-free periods before and after the growing season). Specifically, the 10 and 90% thresholds in the daily gcc between May 1 and the timing of peak gcc were used to define the transition dates from dormancy to green-up in spring and from green-up to peak season, respectively. Meanwhile, the 90 and 10% thresholds between the timing of peak gcc and October 31 were used to define the shifts from peak season to senescence and from senescence to dormancy in autumn, respectively.

### Statistical analysis

The relative importance of the various potential abiotic controls (i.e., including PPFD, WTL, Ta, and Ts at all available depths) in regulating the diel patters in Rh and Ra was examined using a wavelet coherence analysis^58^ of the hourly data. This analysis was complemented by determining the Welch’s cross power spectral density^59^ for those variables that showed strong coherence and lag times of <6 h with Ra or Rh. All data analysis was conducted using the Matlab software (Matlab R2019b, Mathworks, USA).

## Supplementary information

Supplementary Information

Peer Review File

## Data Availability

The data sets collected and analyzed during the current study are publicly available in the Figshare digital repository at [10.6084/m9.figshare.12730949.v1].

## Code availability

Data processing scripts are available upon reasonable request from the corresponding author.
