# Supplementary material for: Bimodal diel pattern in peatland ecosystem respiration rebuts uniform temperature response
Source: Nat Commun. 2020 Aug 26;11:4255. doi: 10.1038/s41467-020-18027-1 (PMC7449960; doi:10.1038/s41467-020-18027-1)
Supplement: Supplementary file 1 — Supplementary Information [file 41467_2020_18027_MOESM1_ESM.pdf]

Supplementary Information for

Bimodal diel pattern in peatland ecosystem respiration rebuts uniform temperature response

by Järveoja et al.

Supplementary Figures

Supplementary Fig. 1: Diel patterns of ecosystem respiration measured by autochambers in 2015-2017

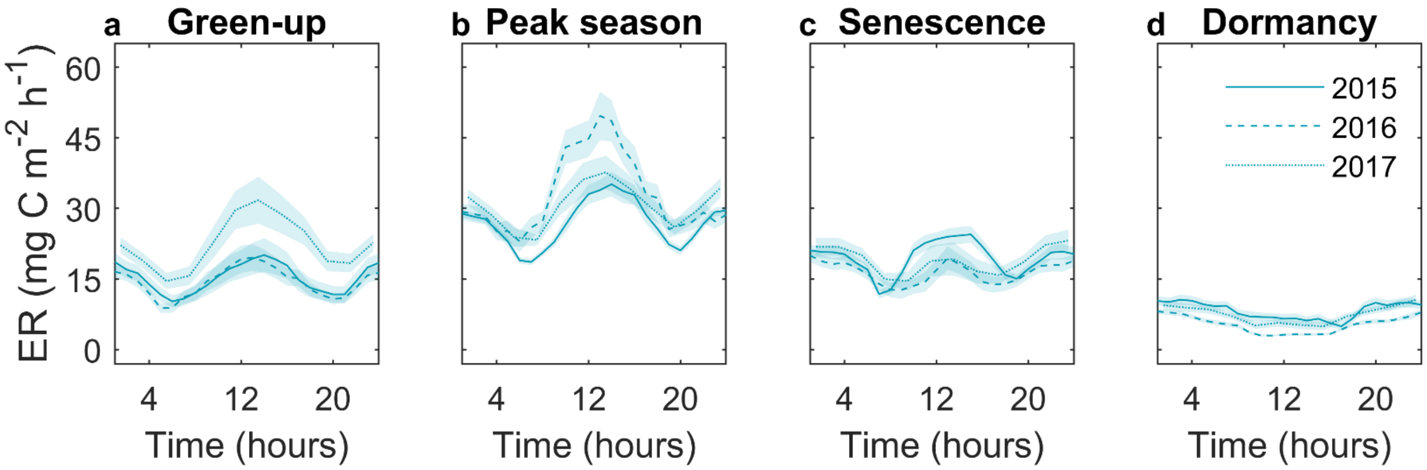

Supplementary Fig. 1: Mean diel patterns of ecosystem respiration (ER) measured by autochambers during the key phenophases of **a** green-up, **b** peak season, **c** senescence and **d** dormancy (spring and autumn) in 2015-2017. Shaded bands indicate ±1 standard error for a given hour resulting from the variation within each phenophase.

Supplementary Fig. 2: Diel patterns of ecosystem respiration derived from eddy covariance measurements in 2015-2017

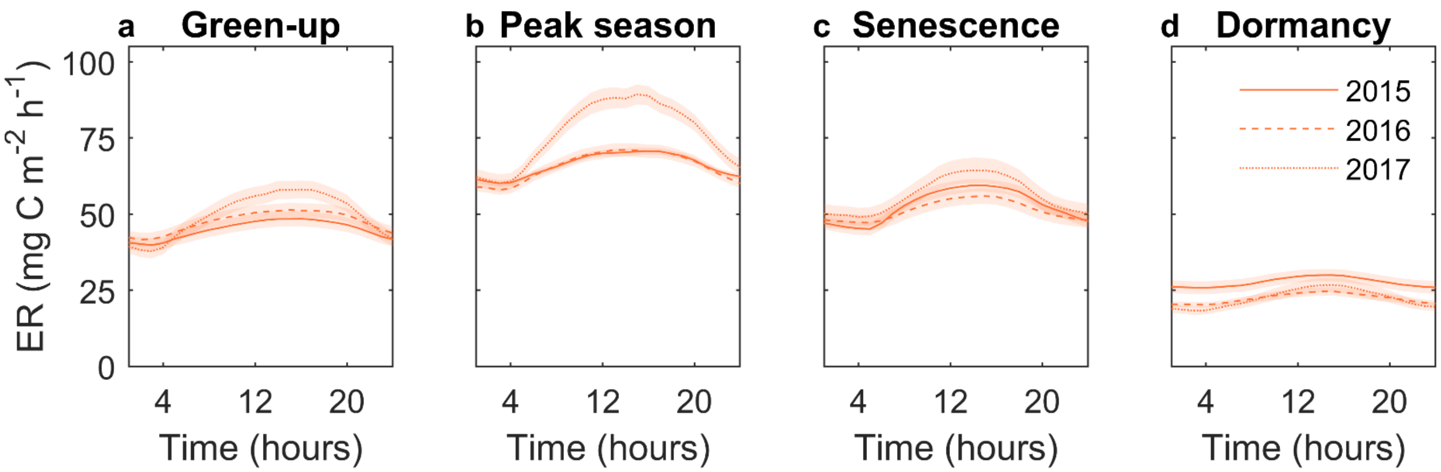

Supplementary Fig. 2: Mean diel patterns of ecosystem respiration (ER) derived from eddy covariance measurements at the ICOS-Degerö station during the key phenophases of **a** green-up, **b** peak season, **c** senescence and **d** dormancy (spring and autumn) in 2015-2017. Shaded bands indicate ±1 standard error for a given hour resulting from the variation within each phenophase.

Supplementary Fig. 3: Hourly bias in extrapolated ecosystem respiration estimates in 2015-2017

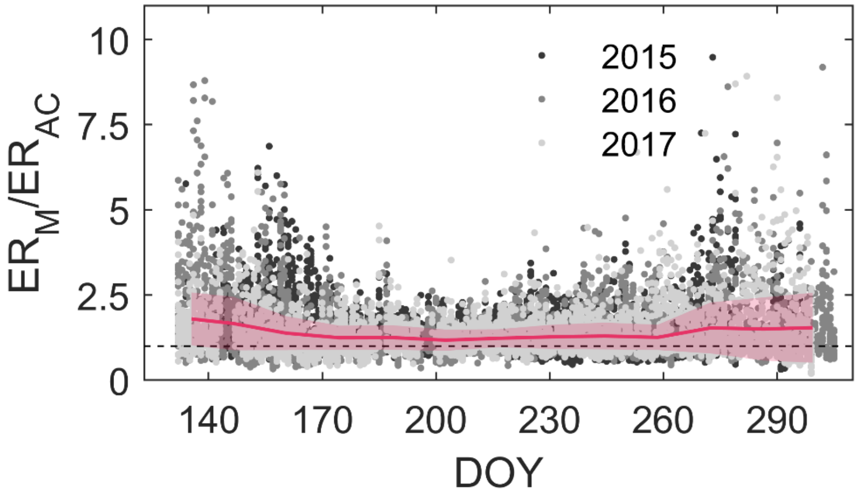

Supplementary Fig. 3: Ratios of hourly ecosystem respiration (ER) estimated by a standard flux modeling approach (ER_M; see Methods for details) and measured by autochambers (ER_{AC}) for the years 2015-2017. Symbols indicate ratios of hourly ER_M and ER_{AC}; the red line represents the block-average (window size = 14 days) with shaded bands indicating ±1 standard error. Horizontal dashed line represents unity of the ratio. The 3-year means of the phenophase transition dates (DOY; day of year) are as follows: 143 (spring dormancy → green-up), 187 (green-up → peak season), 218 (peak season → senescence) and 272 (senescence → autumn dormancy).

Supplementary Fig. 4: Cumulative sums of measured and extrapolated ecosystem respiration in 2015-2017

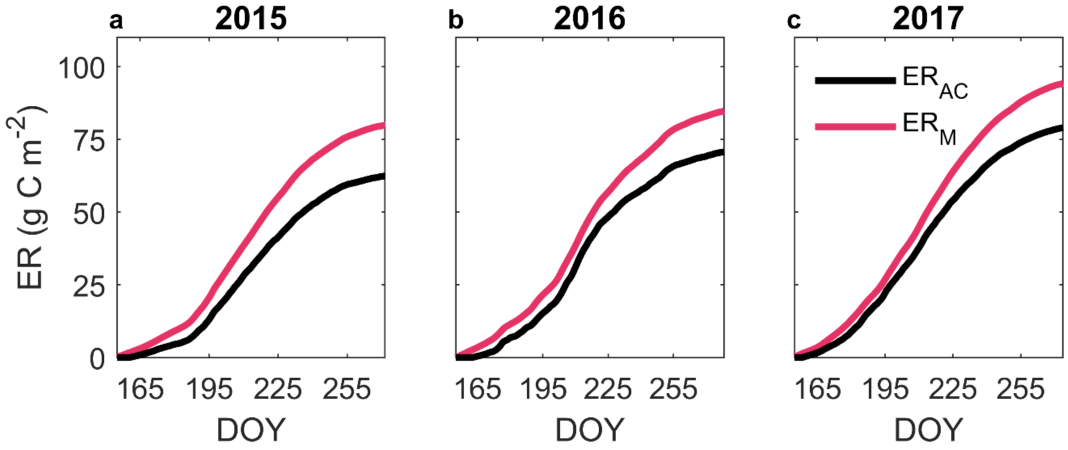

Supplementary Fig. 4: Cumulative sums of ecosystem respiration (ER) measured by autochambers (ER_{AC}) and estimated by a standard flux modeling approach (ER_M; see Methods for details) over the measurement periods of **a** 2015, **b** 2016 and **c** 2017. DOY stands for day of year.

Supplementary Fig. 5: Diel divergence in the temperature response of ecosystem respiration in 2015-2017

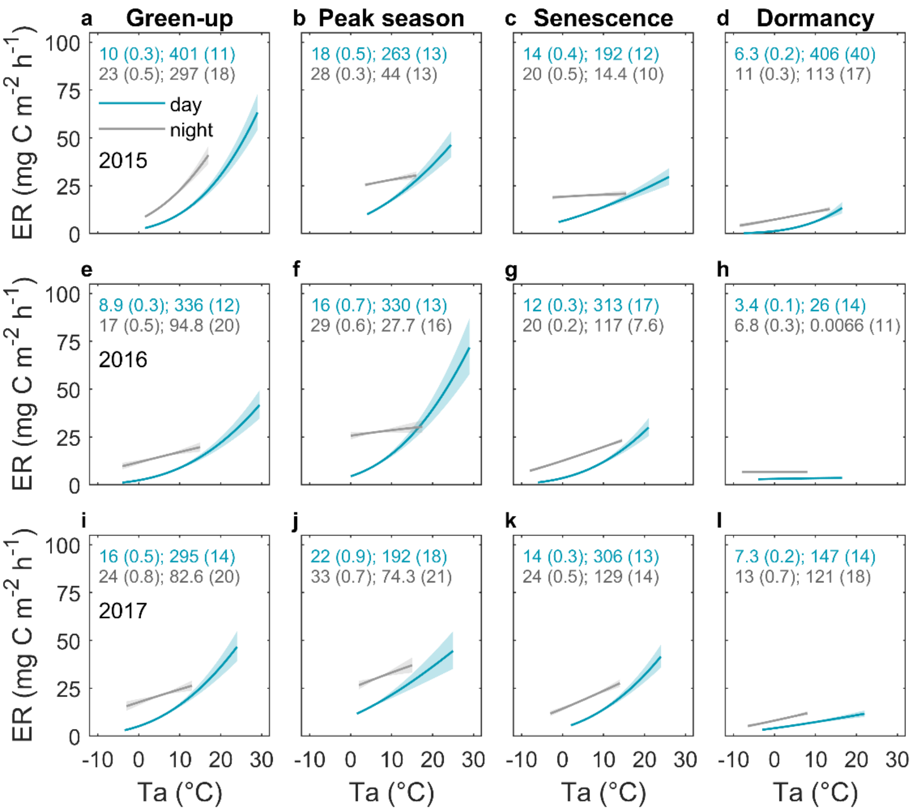

Supplementary Fig. 5: Exponential regression relationships between ecosystem respiration (ER) measured by autochambers and air temperature (Ta) for day- and nighttime (i.e. photosynthetic photon flux density ≥ 20 and < 20 μmols m^{-2} s^{-1}, respectively) during the key phenophases of green-up, peak season, senescence and dormancy (spring and autumn) in **a-d** 2015, **e-h** 2016 and **i-l** 2017. Values shown in the panels (blue and grey for day- and nighttime periods, respectively) represent model parameters R_{10} and E_0 with standard errors in brackets from the Lloyd & Taylor (1994) respiration model (see equation in Methods). Solid lines indicate the exponential fit and shaded bands indicate the 95% confidence intervals.

Supplementary Fig. 6: Diel patterns of heterotrophic and autotrophic respiration in 2015-2016

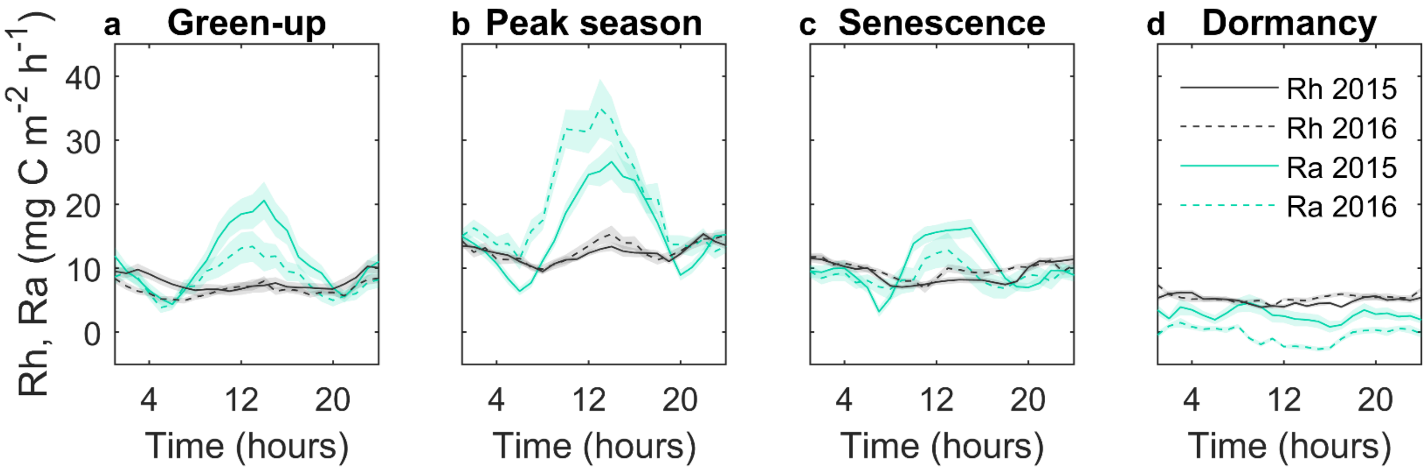

Supplementary Fig. 6: Mean diel patterns of heterotrophic respiration (Rh) and autotrophic respiration (Ra) during the key phenophases of **a** green-up, **b** peak season, **c** senescence and **d** dormancy (spring and autumn) in 2015 and 2016 (no Rh and Ra data were available in 2017). Shaded bands indicate ±1 standard error for a given hour resulting from the variation within each phenophase.

Supplementary Fig. 7: Abiotic controls of heterotrophic and autotrophic respiration in 2016

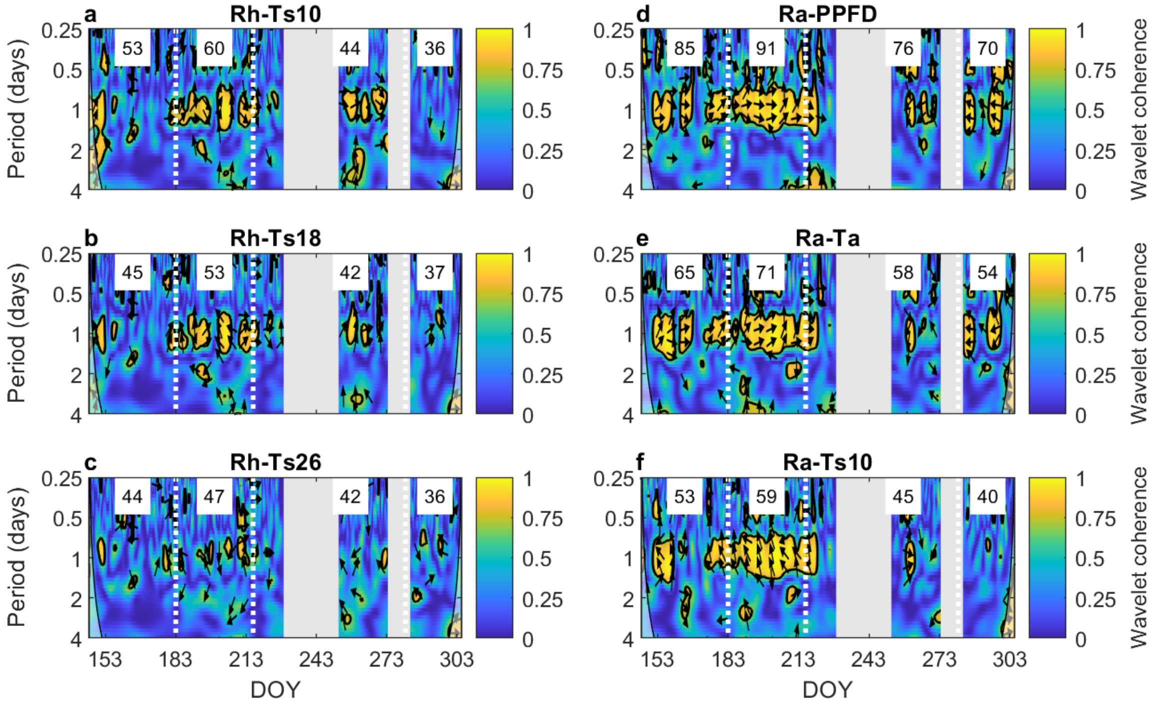

Supplementary Fig. 7: Wavelet coherence (yellow = strong and blue = absent) between hourly fluxes of **a-c** heterotrophic respiration (Rh) and **d-f** autotrophic respiration (Ra) and their abiotic controls (i.e. soil temperature at 10, 18 and 26 cm depth (Ts10, Ts18 and Ts26), photosynthetic photon flux density (PPFD) and air temperature (Ta)) in 2016. Arrows indicate lag: right = no lag, up or down = quarter diel cycle (i.e. 6 hrs) lag of the flux behind or ahead of environmental variable, respectively, left = antiphase lag (i.e. 12 hrs). Grey vertical bands indicate extended periods with missing hourly Rh and Ra data. Vertical dotted lines indicate transitions between the phenophases (green-up → peak season → senescence → autumn dormancy). Numbers in white boxes represent the Welch's cross power spectral density values between the hourly flux and environmental variables at the 1-day period (see also Supplementary Fig. 9). DOY stands for day of year.

Supplementary Fig. 8: Air temperature and water table level effects on heterotrophic respiration in 2015-2016

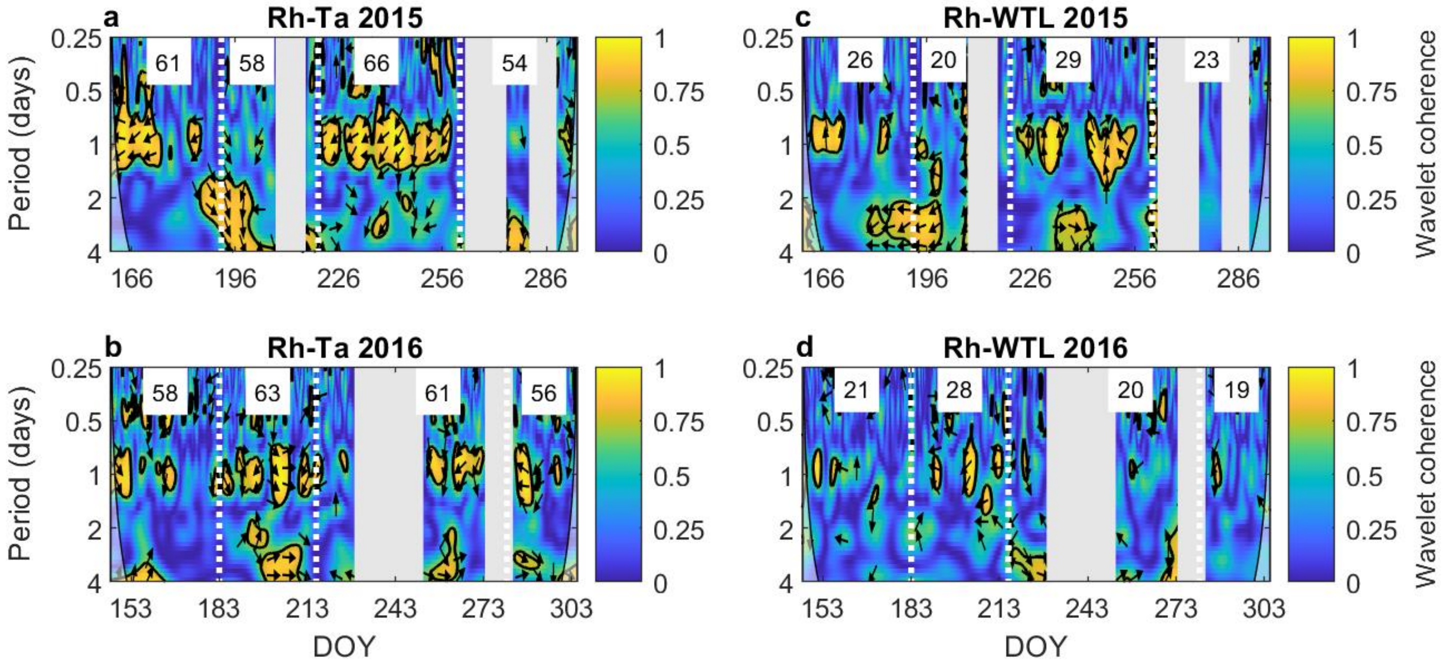

Supplementary Fig. 8: Wavelet coherence (yellow = strong and blue = absent) between hourly heterotrophic respiration (Rh) and **a-b** air temperature (Ta) or **c-d** water table level (WTL) in 2015 and 2016. Arrows indicate lag: right = no lag, up or down = quarter diel cycle (i.e. 6 hrs) lag of Rh behind or ahead of the environmental variables, respectively, left = antiphase lag (i.e. 12 hrs). Grey vertical bands indicate extended periods with missing hourly Rh data. Vertical dotted lines indicate transitions between the phenophases (green-up → peak season → senescence → autumn dormancy). Numbers in white boxes represent the Welch's cross power spectral density values between the hourly flux and environmental variables at the 1-day period. DOY stands for day of year.

Supplementary Fig. 9: Welch's cross power spectral density between hourly heterotrophic and autotrophic respiration and key abiotic controls

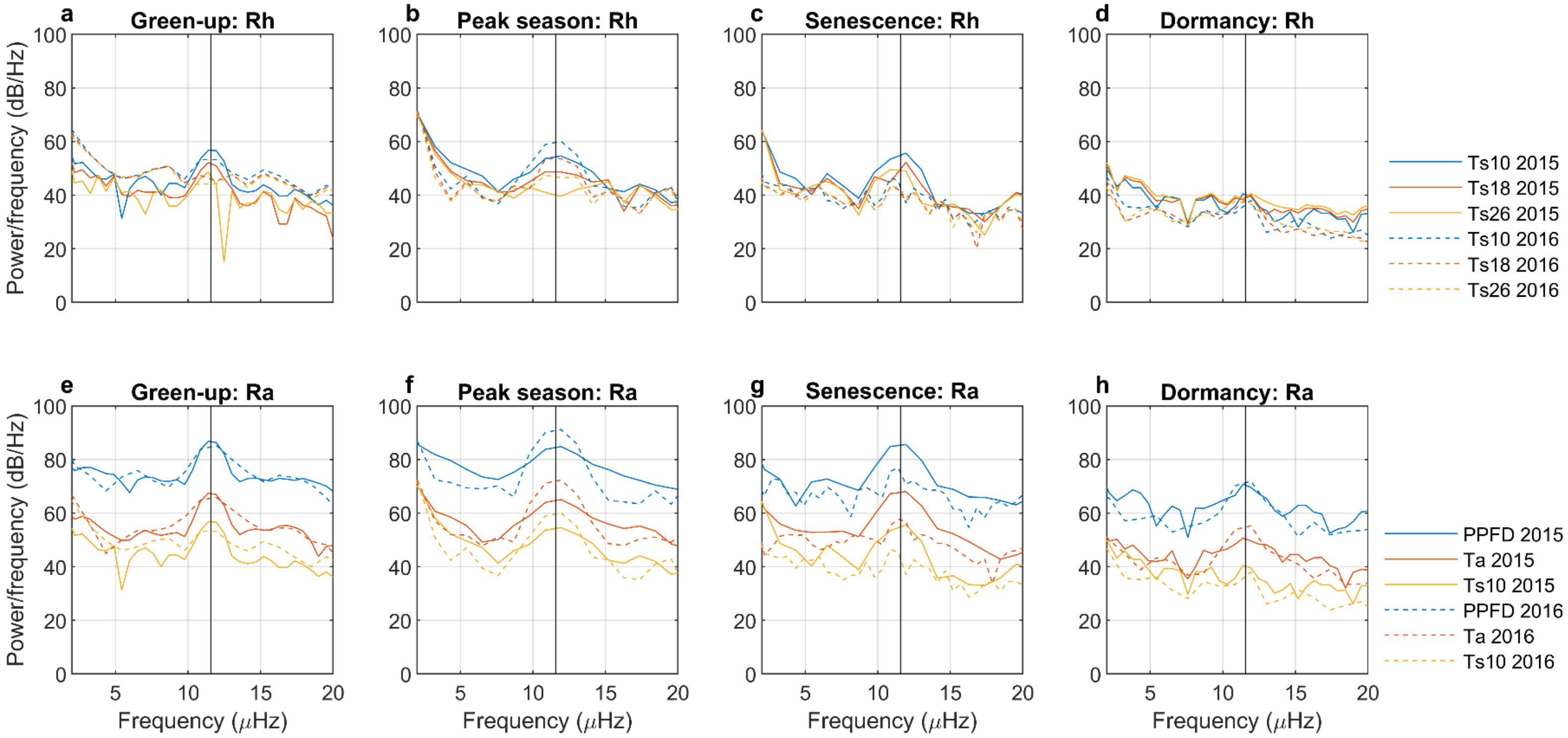

Supplementary Fig. 9: Welch's cross power spectral density between hourly **a-d** heterotrophic respiration (Rh) or **e-h** autotrophic respiration (Ra) and soil temperature at 10, 18 and 26 cm depths (Ts10, Ts18 and Ts26), photosynthetic photon flux density (PPFD) and air temperature (Ta) during the key phenophases of green-up, peak season, senescence and dormancy (spring and autumn) in 2015 (solid lines) and 2016 (dashed lines). Black vertical lines indicate the frequency of 11.57 μHz which corresponds to the diel cycle (i.e. the 1-day period).

Supplementary Fig. 10: Diel patterns of heterotrophic and autotrophic respiration during dry and wet periods

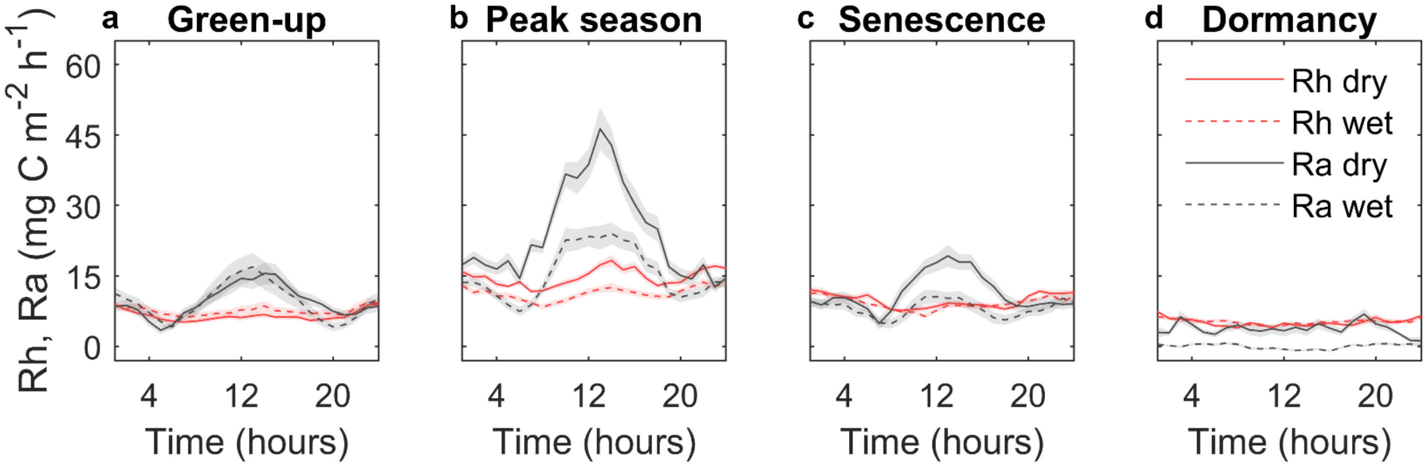

Supplementary Fig. 10: Mean diel patterns of heterotrophic respiration (Rh) and autotrophic respiration (Ra) in the key phenophases of **a** green-up, **b** peak season, **c** senescence and **d** dormancy (spring and autumn) shown as a mean of the years 2015-2016. Data are separated into periods with dry (i.e. water table level < mean water table level) and wet (i.e. water table level ≥ mean water table level) conditions. Shaded bands indicate ±1 standard error for a given hour resulting from the variation within each phenophase and across the measurement years. Note that this analysis does not resemble a comparison of true treatments measured in parallel but instead compares data from different moments in time when also other controlling factors might differ.

Supplementary Fig. 11: Diel patterns of heterotrophic and autotrophic respiration in the four replicate chamber groups

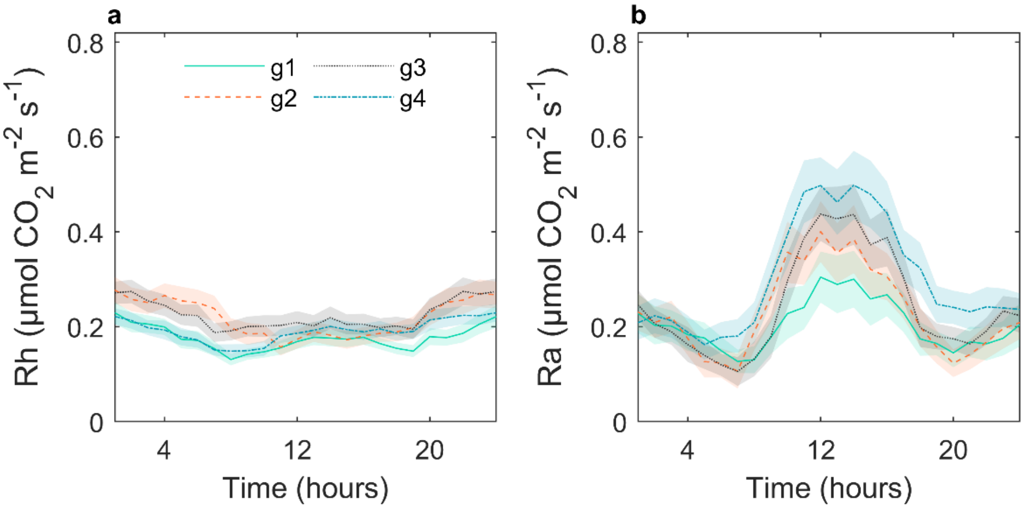

Supplementary Fig. 11: Diel patterns of **a** heterotrophic respiration (Rh) and **b** autotrophic respiration (Ra) in the four replicate chamber groups (i.e. g1, g2, g3 and g4) averaged over the years 2015-2016. To facilitate a direct comparison, this analysis includes only data from periods when at least 3 out of the available 4 chambers were in operation. Across g1 to g4, total vascular plant biomass was estimated at 16, 9, 38 and 54 g m^{-2}, respectively and the corresponding moss area cover at 97.5, 100, 85.5 and 97.5%, respectively.

Supplementary Fig. 12: Diel temperature responses of heterotrophic and autotrophic respiration during wet and dry periods

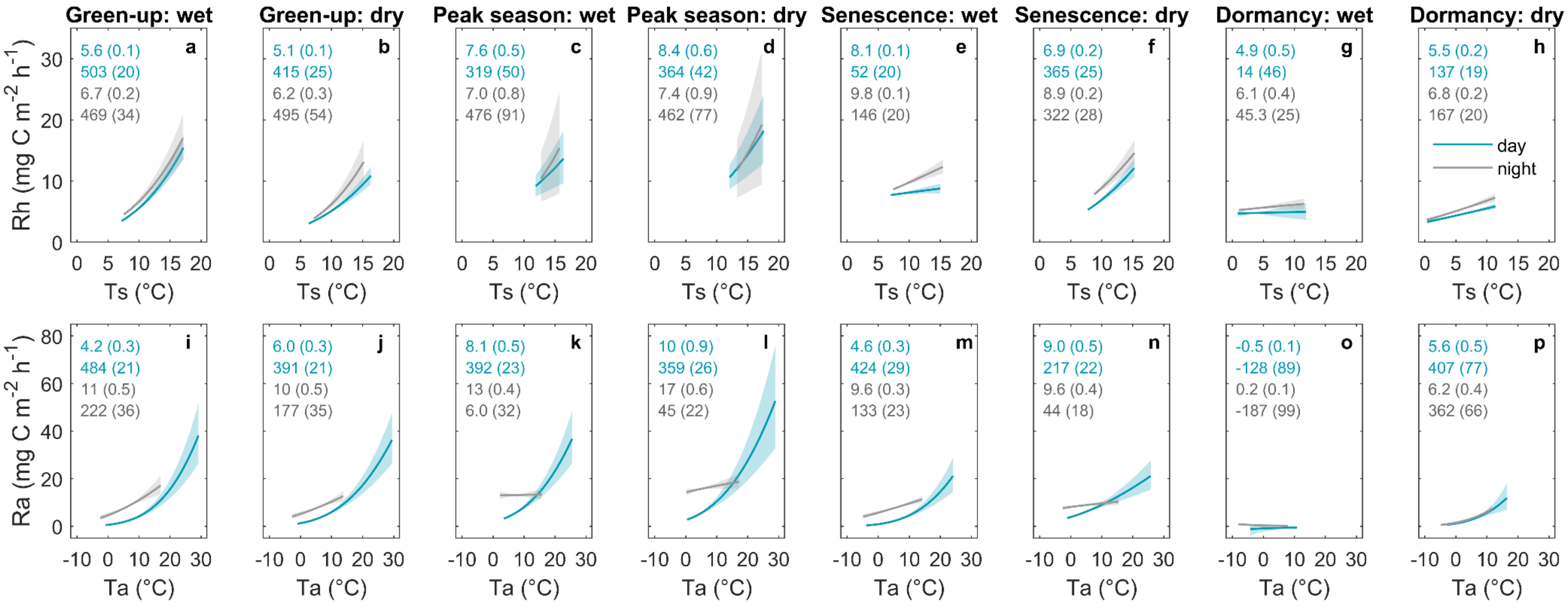

Supplementary Fig. 12: Exponential regression relationships of **a-h** heterotrophic respiration (Rh) and **i-p** autotrophic respiration (Ra) with soil or air temperature (Ts at the 10 cm depth or Ta, respectively) for day- and nighttime (i.e. photosynthetic photon flux density ≥ 20 and < 20 µmols m^{-2} s^{-1}, respectively) during the key phenophases of green-up, peak season, senescence and dormancy (spring and autumn) shown as a mean of the years 2015-2016. Data are separated into periods with dry (i.e. water table level < mean water table level) and wet (i.e. water table level ≥ mean water table level) conditions. Values displayed in the panels (blue and grey for day- and nighttime periods, respectively) represent model parameters R_{10} and E_0 with standard errors in brackets (upper and lower row, respectively, for each day- and nighttime regression) from the Lloyd and Taylor (1994) respiration model (see equation in Methods). Solid lines indicate the exponential fit and shaded bands indicate the 95% confidence intervals.

Supplementary Fig. 13: Effect of an ambient CO_2 concentration filter on the diel patterns of ecosystem respiration measured by autochambers

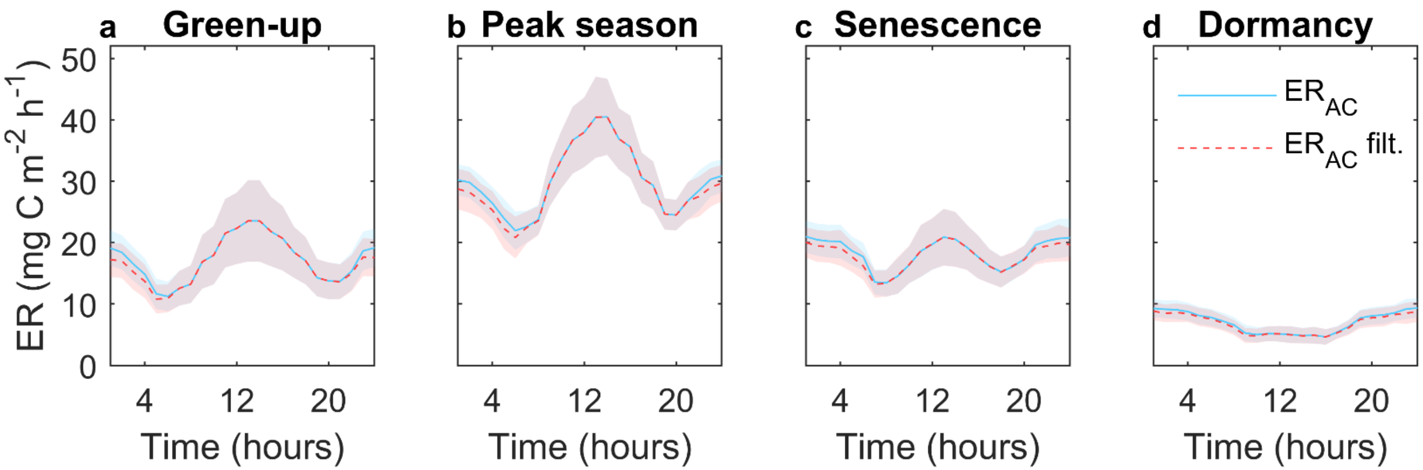

Supplementary Fig. 13: Diel patterns of ecosystem respiration (ER) measured by autochambers during the key phenophases of **a** green-up, **b** peak season, **c** senescence and **d** dormancy (spring and autumn) shown as a mean of the years 2015-2017 before and after filtering with an ambient CO_2 concentration threshold of 415 ppm to eliminate nighttime data during stable atmospheric conditions (ER_{AC} and ER_{AC} filt., respectively). Shaded bands indicate ±1 standard error for a given hour resulting from the variation within each phenophase and across the 3 years.

Supplementary Fig. 14: Diel patterns of the methane flux measured by autochambers

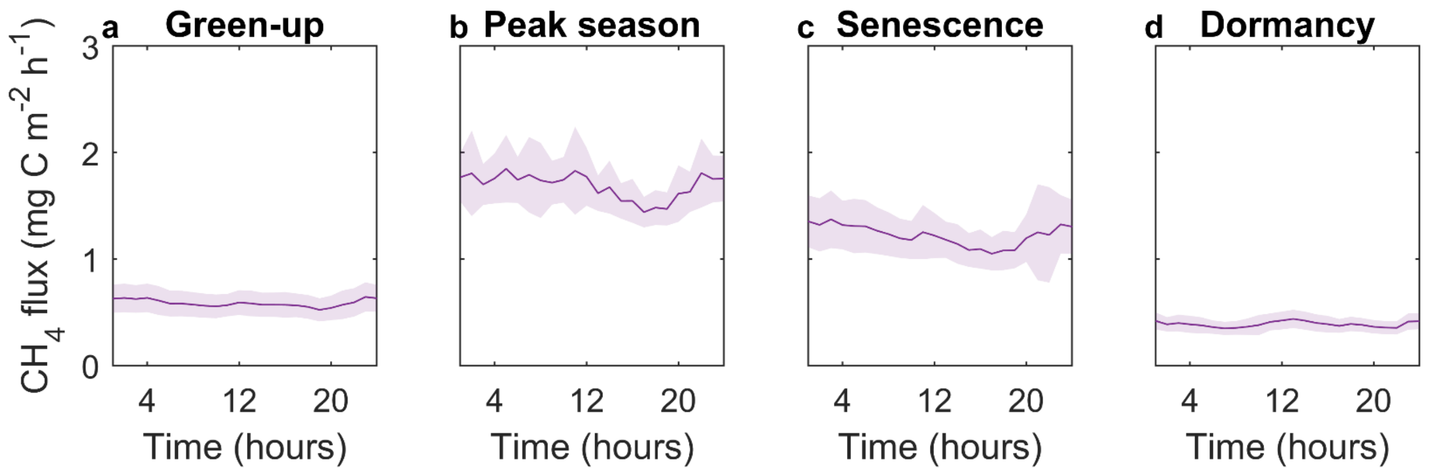

Supplementary Fig. 14: Mean diel patterns of the methane (CH_4) flux measured by autochambers during the key phenophases of **a** green-up, **b** peak season, **c** senescence and **d** dormancy (spring and autumn) shown as a mean of the years 2015-2017. Shaded bands indicate ±1 standard error for a given hour resulting from the variation within each phenophase and across the 3 years.

Supplementary Tables

Supplementary Table 1: Cumulative sums of ecosystem respiration and net CO_2 exchange based on continuous autochamber measurements *versus* extrapolation of daytime data

Supplementary Table 1: Cumulative sums of ecosystem respiration (ER), gross primary production (GPP) and net CO_2 exchange (NEE) for the key phenophases (i.e. green-up, peak season, senescence and dormancy) and the snow-free season averaged over 2015-2017. In the Model estimate, hourly ER is simulated using the daytime parameters (during both day- and nighttime) from the Lloyd & Taylor (1994) respiration model (see equation in Methods) built on the relationships between measured autochamber ER and air temperature (see parameter values in Fig. 3 of the main article). In the AC estimate, ER represents the hourly measured data (during both day- and nighttime) from the dark autochambers. GPP is derived from the difference between the modeled (in Model) or measured (in AC) daytime ER and the daytime NEE measured concurrently with transparent autochambers. Daily NEE is obtained from the sum of measured daytime NEE and either modeled (Model) or measured (AC) nighttime ER (which equals NEE in the absence of photosynthesis). Note that the aim of this analysis was to investigate the potential bias from extrapolating manual daytime dark chamber measurements to the diel and seasonal scales using constant ER-Ta relationships during both day- and nighttime. We therefore assume that manual chamber measurements were conducted during each daytime hour in order to exclude additional confounding effects due to the relatively lower sampling frequency inherent to the manual chamber technique.

	**Green-up**		**Peak season**		**Senescence**		**Dormancy**		**Snow-free season**	
	**Model**	**AC**	**Model**	**AC**	**Model**	**AC**	**Model**	**AC**	**Model**	**AC**
**ER**	12.6	14.3	16.0	17.8	14.7	18.7	2.7	4.4	46.0	55.2
**GPP**	-29.0	-28.9	-37.2	-37.2	-33.0	-33.2	-3.1	-3.1	-102.4	-102.5
**NEE**	-16.4	-14.6	-21.2	-19.4	-18.3	-14.5	-0.4	1.3	-56.4	-47.3
**NEE_{diff}**	**1.8 g (12%)**		**1.8 g (9%)**		**3.8 g (26%)**		**1.7 g (131%)**		**9.1 g (19%)**	
