# Supplementary material for: Bimodal diel pattern in peatland ecosystem respiration rebuts uniform temperature response
Source: Nat Commun. 2020 Aug 26;11:4255. doi: 10.1038/s41467-020-18027-1 (PMC7449960; doi:10.1038/s41467-020-18027-1)
Supplement: Supplementary file 2 — Peer Review File [file 41467_2020_18027_MOESM2_ESM.docx]

Peer Review Information

**Journal:** Nature Communications

**Manuscript title:** Bimodal diel pattern in peatland ecosystem respiration rebuts uniform temperature response

**Corresponding author name(s):** Dr Jarvi Jareoja

**Editorial notes:**

**Transferred manuscripts**

This manuscript has been previously reviewed at another journal that is not operating a transparent peer review scheme. This document only contains reviewer comments and rebuttal letters for versions considered at *Nature Communications*.

**Reviewer comments & decisions:**

**Reviewer comments, first version:**

Reviewers' comments:

Reviewer #1 (Remarks to the Author):

The authors used an automated dark chamber system to investigate the diel dynamics of heterotrophic (Rh) and autotrophic (Ra) respiration in a peatland (fen). Their results are novel (for peatlands) and they question the long-held assumption that these respiration rates follow a uniform diel response to temperature. Their experiment, which was performed over three years, shows divergent temperature controls on day vs night fluxes. They attribute these differences to the relative roles of Rh vs Ra during the diel cycle. They also show interesting peaks in respiration mid-day and mid-night. Importantly, their results clearly show (in my opinion) that the classic "uniform" modeling assumption overestimates daily respiration by ~ 2 times, and growing season respiration by up to about 25%. This implies that models are overestimating peat-C loss to the atmosphere (CO2 from respiration) by a lot!

The manuscript is well written, relatively easy to follow, and the references cited are well chosen. The Method is sound, the discussion is reasonable and honest, and the results/conclusions are important for ecologists, biogeochemists, and modelers interested in peatland ecosystems specifically, but also terrestrial ecosystems in general, as well as the global carbon cycle.

I recommend publication of this manuscript, though I would appreciate if the authors would comment on an aspect that seems neglected in the current study: can you tell us more about the hydrology of the system? Its potential role in controlling Rh and Ra? Or are the intricacies between soil temp and moisture content to complex to fully distantangle? either way, it would be good to provide additional information on the importance of soil moisture content and water table depth on your results. This is particularly important given the minerotrophic conditions you describe.


Reviewer #2 (Remarks to the Author):

This study investigated the temporal patterns in ecosystem respiration (ER) and its components (heterotrophic [Rh] and autotrophic [Ra] respiration) in a peatland. Using continuous (i.e., hourly) measurements of NEE, ER, and Rh over 3 years, the authors found a bimodal response of daily ER, the strength of which varied by season. This bimodal response is in contrast to traditional measurements of ER which assume a unimodal response of ER over a day. Ultimately, the authors concluded that traditional measurements of ER may overestimate ER by 16-23% over a growing season, which has important implications for understanding peatland C fluxes (i.e., sink vs source).

Overall, this is a well-designed and comprehensive study, and the manuscript is very well written. This paper will be impactful to the field, given the importance of accurately quantifying C fluxes from C-rich peatlands under natural and impacted (e.g., drained peatlands, logged peatlands, experimental manipulations to investigate climate change impacts) conditions. While this is the first study to investigate partitioning of ER in peatlands, this approach has been applied to other ecosystems (forests, grasslands; these are cited in the paper), and thus the novelty to justify publication in a high-impact journal such as Nature Communications rather than a more specified journal (e.g., JGR Biogeosciences) is not clear. Given that this is a well-studied peatland, if peatland C flux data are available to help place these ER results into context (i.e., how much does the overestimation of ER affect net C flux estimates from peatlands?), this might increase the impact of this study.

Please see below for specific comments to help clarify some aspects of the paper.

L21 (and L53): Not clear what “respectively” is referring to here – do you mean eddy covariance data extrapolate nighttime data and manual chambers extrapolate daytime data?

L98: I assume this range of 16-23% represents the range measured over those 3 years?

L108: This section focuses on the temporal controls on Rh and Ra; however, the Introduction mentions the importance of both spatial and temporal variation (L46). While I understand an investigation of spatial variation (e.g., differences across microtopographies, across different peatlands, etc) is out of the scope of this study, I wonder how variable the diel patterns in Rh and Ra are across the replicate chambers. Was Rh or Ra more variable across space? If Ra was variable, were there differences in vegetation composition across chambers that could explain some of this variation?

L123-125: Were other potential controls on seasonality in Rh besides temperature examined? (i.e., water table dynamics).

L146-148: Given that net C flux estimates have been conducted in this peatland in the past (Nilsson et al. 2008), can you estimate the degree to which an estimate of peatland net C flux may be affected by the overestimation of ER using traditional methods?

L182-185: However, wasn’t the magnitude of Ra larger than that of Rh (i.e., Figure 4)? Unless I’m not interpreting correctly, this sentence seems to highlight the magnitude of Rh over Ra.

L244: By “recent root exudates”, do you mean exudates that were released during trenching? The trenching was done almost 2 years before measurements started, but was there any evidence of a disturbance effect from the trenching on respiration?

L250: Suggest providing the area of the chamber earlier in the Methods (i.e., L227).

L257: Check spelling on “chamber”.

L268: Is this climate station in the mire itself or elsewhere?

L267-268: I believe that relationships between Rh and Ra vs soil temperature were only investigated for a few of these depths (10, 18, 26 cm). What were the temperature data measured at the other depths used for?

L275-276: Were the measurements across the 12 chambers taken at random over the hour, or were they sequential (i.e., all the clear chambers measured first, then all the dark chambers, then dark trenched chambers)?

L490: What does the standard error represent? Variation across years? Across replicate chambers?

L513-524: What do the colors (blue to yellow) represent?

Figure 1 – It would be helpful to indicate when day vs night is on these figures as it changes over the season (i.e., include grey shading over nighttime hours).


Reviewer #3 (Remarks to the Author):

Comments to author:

The authors present claims on a diel pattern in the temperature response of soil and ecosystem respiration on a nothern undrained peatland based on a semi-long-term measurement campaign. The manuscript is well-written, to the point and concise. The authors base their claims on patterns evident in the measured data. If valid, the results will significantly improve estimates of ecosystem respiration on peatlands and thus aid in the effort to predict the present and future carbon balance of peatlands.

However, the authors do not present convincing evidence that the observed pattern (ie. higher base rate and lower temperature response of respiration during the night vs. during the day) is not an artefact caused by accumulation of CO2 into the soil surface during night, which has been observed to cause erroneously high CO2 emission estimates on peatlands in other studies. My recommendation would be to recognize the time periods when this has occurred during the campaign, remove the measurements affected from the analysis and see if the diel pattern still exists.

I am also not convinced by the authors' reasoning as to the mechanistic cause of the high night-time respiration and would like to see more discussion on the importance of permanently inundated peat layers to the total ecosystem respiration.

Specific comments follow:

L56-57 This claim would benefit from a couple of references

L99-101 The Lloyd-Taylor paper discussing exponential models of ER is referenced in this manuscript; why is the improved exponential respiration model presented in that paper used here?

L111-113 How sure are you that the night-time peak is not due to accumulation of CO2 in the surface layer during still clear nights during the summer?

L121-125 This relatively weak correlation leaves me unconvinced that such a large effect on Rh could be caused by the rather small variations in soil temperatures. I would like to see the measured temperatures in a plot of some kind of deviation from the daily mean per layer and how those deviations occur over the course of the day, on average over the measurement campaign.

L175-177 The peat layers below 13 cm are permanently inundated in this bog, according to Fig. 1 in Järveoja et al. 2018. How can the temperature in these layers affect ER as measured in CO2 emission in this magnitude (10 mg C m-2 h-1, re Fig. 1 in this manuscript)?

L256-257 This measurement period is very, very short! It makes the measurements sensitive to initial disturbances to the soil surface caused by the closing of the chamber and changing turbulence conditions.

L287-290 I'm not entirely convinced by the procedure presented in Järveoja et al. 2018, in that the correction term seems very uncertain related to the data points presented. I'd like to see what effect removing the measurements where CO2 accumulation on the surface layer is an issue would have on the night-time Rh and Ra temperature dependency.

**Author rebuttal, first version:**

**REVIEWER 1:**

The authors used an automated dark chamber system to investigate the diel dynamics of heterotrophic (Rh) and autotrophic (Ra) respiration in a peatland (fen). Their results are novel (for peatlands) and they question the long-held assumption that these respiration rates follow a uniform diel response to temperature. Their experiment, which was performed over three years, shows divergent temperature controls on day vs night fluxes. They attribute these differences to the relative roles of Rh vs Ra during the diel cycle. They also show interesting peaks in respiration mid­day and mid-night. Importantly, their results clearly show (in my opinion) that the classic "uniform" modeling assumption overestimates daily respiration by ~ 2 times, and growing season respiration by up to about 25%. This implies that models are overestimating peat-C loss to the atmosphere (CO2 from respiration) by a lot!

The manuscript is well written, relatively easy to follow, and the references cited are well chosen. The Method is sound, the discussion is reasonable and honest, and the results/conclusions are important for ecologists, biogeochemists, and modelers interested in peatland ecosystems specifically, but also terrestrial ecosystems in general, as well as the global carbon cycle.

I recommend publication of this manuscript, though I would appreciate if the authors would comment on an aspect that seems neglected in the current study: can you tell us more about the hydrology of the system? Its potential role in controlling Rh and Ra? Or are the intricacies between soil temp and moisture content to complex to fully distantangle? either way, it would be good to provide additional information on the importance of soil moisture content and water table depth on your results. This is particularly important given the minerotrophic conditions you describe.

**Response: We appreciate the positive feedback from the reviewer on our manuscript. We have followed up on the question about the hydrology of the mire system by providing more information on water table level (WTL) dynamics in the Methods section (L284-286). Specifically, we provide information on the snow-free season range and mean of the WTL variation: ‘*The water table level relative to the mire surface within our measurement area ranges between 0 and -15 cm with a snow-free season mean of -4.7 cm (Järveoja et al. 2018)*’. Furthermore, we now clarify already at the end of the Introduction section that our study site is a ‘*minerogenous*’ peatland, i.e. a fen (L69).**

**We also appreciate the reviewer’s suggestion on exploring soil moisture effects on our results in more detail. Unfortunately, we do not have well calibrated soil moisture data available for additional analysis. However, a strong relationship between WTL and soil moisture in the unsaturated zone has previously been reported for this same mire site by Nijp et al. (2017). We have previously investigated the wavelet coherence of Rh and Ra with WTL, though not presented**

1

**Järveoja et al. Authors’ response to reviewers’ comments**

**it in the manuscript since the coherences were weak relative to the other controlling variables (see Fig. R1 below).**

**Fig. R1. Wavelet coherence (yellow = strong and blue = absent) between hourly *a-b* heterotrophic
(Rh) and *c-d* autotrophic respiration (Ra) with water table level (WTL) in 2015 and 2016. Arrows
indicate lag: right = no lag, up or down = quarter diel cycle (i.e. 6 hrs) lag of flux behind or ahead of
WTL, respectively, left = antiphase lag (i.e. 12 hrs). Grey vertical bands indicate extended periods
with missing hourly Rh data. Vertical dotted lines indicate transitions between the phenophases
(green-up -* peak season -* senescence -* autumn dormancy). Numbers in white boxes represent
the Welch’s cross power spectral density values between the hourly flux and WTL at the 1-day
period. DOY stands for day of year.**

**However, encouraged by the reviewers comment we have made an additional analysis to explore the role of WTL on controlling i) the diel patterns and ii) the temperature responses of the respiration fluxes (Figs. R2 and R3). For this purpose, we separated our flux data into periods when the WTL was above or below the mean WTL (referred to as ‘wet’ and ‘dry’, respectively, hereafter) for each of the phenophases.**

**The results from this additional analysis suggest similar bimodal diel patterns for ER, Rh and Ra during both wet and dry conditions (Fig. R2), although the fluxes are consistently higher during the dry periods, particularly in the peak season. Moreover, we find that dry conditions enhance primarily the daytime ER fluxes due to an increase in Ra. It is further noteworthy that the Ra increase during dry conditions is more pronounced during daytime while the response of diel Rh to WTL is similar for day- and nighttime conditions. We further find that WTL effects (compare panels ‘wet’ and ‘dry’ in Fig. R3) on both parameters describing the response to soil temperature (Ts; 10 cm depth) in the Lloyd & Taylor (1994) respiration model (i.e. R10, base respiration at 10 °C and E0, activation energy) are generally smaller for Rh compared to Ra. Overall, these results show that WTL variations have a greater effect on both diel patterns and temperature responses of Ra compared to Rh. We have incorporated the additional effects of WTL on the dynamics of the respiration fluxes in the Results and Discussion sections of the revised manuscript (L139-142, L160-**

2

Järveoja et al. Authors’ response to reviewers’ comments

**162, L171ff) and also included Figs. R1a-b, R2 and R3 as part of the Supplementary material (Supplementary Figs. 8c-d, 10 and 12).**

**Fig. R2. Mean diel patterns for *a-d* heterotrophic (Rh) and autotrophic respiration (Ra) in the key phenophases of green-up, peak season, senescence and dormancy (spring and autumn) in 2015­2016. Data are separated into periods with dry (i.e. water table level < mean water table level) and wet (i.e. water table level ≥ mean water table level) conditions. Shaded bands indicate ±1 standard error for a given hour resulting from the variation within each phenophase and across**

**the measurement years.**

**Fig. R3. Exponential regression relationships of *a-h* heterotrophic (Rh) and *i-p* autotrophic
respiration (Ra) with soil or air temperature (Ts at the 10 cm depth or Ta, respectively) for day-**
**and nighttime (i.e. photosynthetic photon flux density ≥ 20 and < 20 μmols m^-2^ s^-1^, respectively)
during the key phenophases of green-up, peak season, senescence and dormancy (spring and**

**autumn). Data are separated into periods with dry (i.e. water table level < mean water table level)
and wet (i.e. water table level ≥ mean water table level) conditions. Values shown in the panels
(blue and grey for day- and nighttime periods, respectively) represent model parameters R10 and**

**E0 with standard errors in brackets from the Lloyd and Taylor (1994) respiration function** = (

. −

−.). **Solid lines indicate the exponential fit and shaded bands indicate**

**the 95% confidence intervals.**

3

**Järveoja et al. Authors’ response to reviewers’ comments**

**As we further clarify in the revised manuscript (Supplementary Fig. 10), a more detailed interpretation of the underlying causes for the observed variations in the ‘wet’ vs ‘dry’ comparison is, however, too speculative given the imperfect design of this comparison. Specifically, one must recognize that here we do not compare true treatments that were measured in parallel but instead compare data from different moments in time when also other controlling factors might differ. For instance, data obtained during drier periods likely coincide with higher temperature and solar radiation which could further enhance primarily Ra via increased plant production (but also Rh due to its temperature dependency). Thus, as indicated by the reviewer, it is a too complex of a matter to conclusively disentangle the intricacies of soil temperature and moisture as well as plant phenology on the respiration fluxes with our current dataset. We believe that further and more robust insights would require an in-depth investigation with a controlled experimental set-up that is unfortunately beyond the scope of this study.**

**Action: We have added more details in the description of the hydrology of the mire in the Introduction (L69) and Methods sections (L284-286). Furthermore, we made an additional analysis illustrating WTL effects on i) the diel patterns and ii) the temperature response of the separate respiration fluxes. We incorporated this analysis into the revised manuscript (L139-142, L160-162, L171ff) and also included the associated figures in the Supplementary material (Supplementary Figs. 8c-d, 10 and 12).**

**Cited references: Järveoja et al. (2018) Partitioning of the net CO2 exchange using an automated chamber system reveals plant phenology as key control of production and respiration fluxes in a boreal peatland. Global Change Biology 24:3436–3451,** [**https://doi.org/10.1111/gcb.14292**](https://doi.org/10.1111/gcb.14292)

**Nijp et al. (2017) Including hydrological self-regulating processes in peatland models: Effects on peatmoss drought projections. Science of the Total Environment 580:1389–1400,** [**https://doi.org/10.1016/j.scitotenv.2016.12.104**](https://doi.org/10.1016/j.scitotenv.2016.12.104)

4

Järveoja et al. Authors’ response to reviewers’ comments

**REVIEWER 2:**

This study investigated the temporal patterns in ecosystem respiration (ER) and its components (heterotrophic [Rh] and autotrophic [Ra] respiration) in a peatland. Using continuous (i.e., hourly) measurements of NEE, ER, and Rh over 3 years, the authors found a bimodal response of daily ER, the strength of which varied by season. This bimodal response is in contrast to traditional measurements of ER which assume a unimodal response of ER over a day. Ultimately, the authors concluded that traditional measurements of ER may overestimate ER by 16-23% over a growing season, which has important implications for understanding peatland C fluxes (i.e., sink vs source).

Overall, this is a well-designed and comprehensive study, and the manuscript is very well written. This paper will be impactful to the field, given the importance of accurately quantifying C fluxes from C-rich peatlands under natural and impacted (e.g., drained peatlands, logged peatlands, experimental manipulations to investigate climate change impacts) conditions. While this is the first study to investigate partitioning of ER in peatlands, this approach has been applied to other ecosystems (forests, grasslands; these are cited in the paper), and thus the novelty to justify publication in a high-impact journal such as Nature Communications rather than a more specified journal (e.g., JGR Biogeosciences) is not clear.

**Response: We acknowledge that automated chamber systems have been used to partition respiration fluxes in other ecosystems. However, it is unlikely that one could infer dynamics and controls of ER and its component fluxes from results obtained in other ecosystems since peatlands are characterized by rather different vegetation communities and soil carbon (C) stocks. Thus, the detailed information for a peatland ecosystem presented in this study is novel and urgently needed. Furthermore, we would like to emphasize that the novelty of our study does not result from only the *application of an automated chamber system to partition the ER flux*. Instead, we argue that the novelty of our study lies in the combination of *the uniqueness of the dataset*, *the results that challenge a long-standing paradigm* and *the far-reaching implications*. Specifically, we present continuous (at hourly resolution) empirical data on the diel patterns of ER and its separate Rh and Ra components which is unprecedented for a northern peatland ecosystem. In addition, our findings on the bimodal diel patterns and non-uniform temperature response of ER, Rh and Ra provide first-time empirical evidence that disputes the current paradigm that assumes a constant temperature response and therefore a unimodal diel pattern of ER for a norther peatland. Today, the global flux community using eddy covariance (EC) and manual chambers fully relies on this long-held assumption for extrapolation and modeling of ER. Thus, our results have large implications for i) field studies as well as for ii) ecosystem modeling and iii) remote sensing communities, with the latter two relying heavily on accurate field data on C cycle component fluxes for validation purposes. In our view, the sum of these novel aspects absolutely justifies publication in a high-impact journal such as Nature Communications. Finally, despite the presence of previous ER partitioning studies conducted in other ecosystems types, we find that there is still a general lack of understanding in the scientific community (particularly in EC studies) on the fact that ER is composed of two fundamentally different fluxes, i.e. Rh and Ra, which are regulated by different controls. We therefore see the publication of our study in this well-respected journal as the most effective channel for increasing awareness within the flux community on the current conceptual shortcomings, which is urgently needed to improve quantitative estimates of terrestrial ecosystem C cycle components.**

5

Järveoja et al. Authors’ response to reviewers’ comments

Given that this is a well-studied peatland, if peatland C flux data are available to help place these ER results into context (i.e., how much does the overestimation of ER affect net C flux estimates from peatlands?), this might increase the impact of this study.

**Response: We have incorporated the reviewer’s suggestion and now place the observed bias in ER into context with the magnitudes of the net CO2 balance (NEE) and with the net ecosystem carbon balance (NECB) previously determined at our mire site. Specifically, we added in the Discussion section that the estimated ER bias over the entire season ER (ranging from 15-17 g C m^-2^ during the 3 measurement years) is considerable since it represents about one quarter of the long-term mean annual NEE of 58±21 g C m^-2^ (Peichl et al. 2014) and about two thirds of the annual NECB of 23.5 g C m^-2^ (Nilsson et al. 2008). Moreover, as explained in our response to the reviewer’s specific comment further below, we have also estimated that using the daytime temperature response parameters for estimating nighttime ER in the manual chamber approach would result in an overestimation of the net CO2 uptake (NEE) during the snow-free season by 19% at our study site (L192-196).**

**Action: We have extended the Discussion section on the potential ER bias impact on the mire C balance in the revised manuscript version (L192-196).**

**Cited references: Peichl et al. (2014) A 12-year record reveals pre-growing season temperature and water table level threshold effects on the net carbon dioxide exchange in a boreal fen. Environmental Research Letters 9: 055006,** [**https://doi.org/10.1088/1748-9326/9/5/055006**](https://doi.org/10.1088/1748-9326/9/5/055006)

**Nilsson et al. (2008) Contemporary carbon accumulation in a boreal oligotrophic minerogenic mire – a significant sink after accounting for all C-fluxes. Global Change Biology 14:2317–2332,** [**https://doi.org/10.1111/j.1365-2486.2008.01654.x**](https://doi.org/10.1111/j.1365-2486.2008.01654.x)

Please see below for specific comments to help clarify some aspects of the paper.

L21 (and L53): Not clear what “respectively” is referring to here – do you mean eddy covariance data extrapolate nighttime data and manual chambers extrapolate daytime data?

**Response: Yes, exactly. We have now revised these sentences for clarity (L19-22 and L50-54).** L98: I assume this range of 16-23% represents the range measured over those 3 years?

**Response: Yes, that is correct. The exact values were 16, 17 and 22% in 2015, 2016 and 2017, respectively. We have clarified this in the manuscript (L102-104). We also noticed that 23% was previously estimated due to a rounding error which we have now corrected to 22%.**

L108: This section focuses on the temporal controls on Rh and Ra; however, the Introduction mentions the importance of both spatial and temporal variation (L46). While I understand an investigation of spatial variation (e.g., differences across microtopographies, across different peatlands, etc) is out of the scope of this study, I wonder how variable the diel patterns in Rh and Ra are across the replicate chambers. Was Rh or Ra more variable across space? If Ra was variable, were there differences in vegetation composition across chambers that could explain some of this variation?

6

**Järveoja et al. Authors’ response to reviewers’ comments**

**Response: Following the reviewer’s comment we have produced a figure displaying the spatial variation of the Rh and Ra diel patterns based on the 4 replicate chamber groups (Fig. R4). It shows that i) spatial variability is generally larger for Ra than for Rh and ii) spatial variation in Ra increases during daytime whereas no clear diel pattern exists in the variation of Rh. The spatial variability in Ra can be related to differences in total plant biomass, i.e. higher plant biomass resulted in greater amplitudes in diel Ra (Fig. R4, Tables R1 and R2). Meanwhile, a species composition effect on the variations in Ra could not be identified conclusively since the chamber with highest Ra (g4) had both the highest contribution from shrubs but also the highest total biomass. Thus, it remains unclear if total biomass or shrub dominance explains its high Ra. Overall, when comparing across all 4 chambers, we conclude that total biomass rather than sedge/shrub composition explains the variation in Ra. Additional spatial differences might occur from variations in microtopography and associated fluctuations in water table level. We further note that the implication from these results is that diel variations in ER are strongly driven by Ra as a function plant biomass.**

**Fig. R4. Diel patterns of a heterotrophic (Rh) and b autotrophic respiration (Ra) in the four
replicate chamber groups (i.e. g1, g2, g3 and g4) averaged over the years 2015-2016. To facilitate a
direct comparison, this analysis includes only data from periods when at least 3 out of the
available 4 chambers were in operation.**

**Table R1. Vascular plant biomass (g m^-2^) within the four replicate chamber groups (i.e. g1 to g4) averaged over the growing seasons of 2015 and 2016**

7

| **Species** |  |  | **ER chambers** | |  |
| --- | --- | --- | --- | --- | --- |
|  | g1 | g2 | g3 | g4 | Mean (SE) |
| *Eriophorum vaginatum* | 6.6 | 0.2 | 8.1 | 0.9 | 4.0 (1.7) |
| *Carex pausiflora* | 0 | 0.01 | 1.4 | 0.2 | 0.4 (0.3) |
| *Trichophorum cespitosum* | 0 | 0 | 0.5 | 0.1 | 0.2 (0.1) |
| *Scheuchzeria palustris* | 2.7 | 3.91 | 7.0 | 12.0 | 6.4 (1.8) |
| *Vaccinium oxycoccos* | 0.8 | 0.9 | 3.6 | 13.3 | 4.7 (2.6) |
| *Andromeda polifolia* | 6.0 | 3.5 | 17.3 | 27.0 | 13.5 (4.7) |
| **Total** | **16.1** | **8.52** | **37.9** | **53.5** | **29.0 (8.9)** |

**Järveoja et al. Authors’ response to reviewers’ comments**

Numbers in parentheses indicate ± one standard error

Modified from Järveoja et al. (2018) Supporting Information, GCB

**Table R2. Area cover (%) of *Sphagnum* mosses within the four replicate chamber groups (i.e. g1 to g4) averaged over the growing seasons of 2015 and 2016**

| **Species** |  |  | **Dark chambers** | |  |
| --- | --- | --- | --- | --- | --- |
|  | g1 | g2 | g3 | g4 | Mean (SE) |
| *S. majus/balticum* | 35 | 12 | 46.5 | 93.5 | 46.8 (14.9) |
| *S. papillosum* | 58.5 | 83.5 | 28.5 | 1.5 | 43.0 (15.4) |
| *S. lindbergii* | 4 | 4.5 | 10.5 | 2.5 | 5.4 (1.5) |
| **Total** | **97.5** | **100** | **85.5** | **97.5** | **95.1 (2.8)** |

Numbers in parentheses indicate ± one standard error

Modified from Järveoja et al. (2018) Supporting Information, GCB

**Action: We have added text into the Results and Discussion sections pointing out that the spatial variability in ER is driven by that of Ra in dependence of plant biomass (L162-165, L259ff). We have also included the Fig. R4 in the Supplementary material of the manuscript (Supplementary Fig. 11).**

**Cited references: Järveoja et al. (2018) Partitioning of the net CO2 exchange using an automated chamber system reveals plant phenology as key control of production and respiration fluxes in a boreal peatland. Global Change Biology 24:3436–3451,** [**https://doi.org/10.1111/gcb.14292**](https://doi.org/10.1111/gcb.14292)

L123-125: Were other potential controls on seasonality in Rh besides temperature examined? (i.e., water table dynamics).

**Response: We can confirm that we have examined all potentially relevant controls on Rh including WTL but only presented those with highest coherences. Below is a figure showing the wavelet coherence between hourly Rh and WTL (Fig. R5). The Welch’s cross power spectral density values for Rh and WTL ranged between 19 and 29 during the various phenophases in 2015 and 2016 which is considerably lower compared to other drivers. We have clarified which potential drivers were considered in the analysis in the revised manuscript (Methods section L414-416), included the result on the Rh-WTL wavelet coherence (Results section L125-130) and included Fig. R5 as part of the Supplementary material (Supplementary Fig. 8c-d).**

8

**Järveoja et al. Authors’ response to reviewers’ comments**

**Fig. R5. *a-b* Wavelet coherence (yellow = strong and blue = absent) between hourly heterotrophic respiration (Rh) with water table level (WTL) in 2015 and 2016. Arrows indicate lag: right = no lag, up or down = quarter diel cycle (i.e. 6 hrs) lag of Rh behind or ahead of WTL, respectively, left = antiphase lag (i.e. 12 hrs). Grey vertical bands indicate extended periods with missing hourly Rh data. Vertical dotted lines indicate transitions between the phenophases (green-up -* peak season -* senescence -* autumn dormancy). Numbers in white boxes represent the Welch’s cross power spectral density values between the hourly Rh and WTL at the 1-day period. DOY stands for day of**

**year.**

L146-148: Given that net C flux estimates have been conducted in this peatland in the past (Nilsson
et al. 2008), can you estimate the degree to which an estimate of peatland net C flux may be
affected by the overestimation of ER using traditional methods?

**Response: We agree with the reviewer that understanding the impact of biased ER estimates on the peatland net CO2 balance (NEE) is of high interest. However, we note that such evaluation is not straightforward. Specifically, the impact would vary across different flux measurement approaches. For instance, EC studies estimate NEE directly and missing data is gap-filled with look­up tables built on relationships between measured NEE and abiotic variables. Thus, annual NEE sums are independent from the annual ER model estimate. In comparison, annual NEE sums in manual chamber studies are derived from the difference between modeled annual ER and GPP. Thus, here the bias in upscaling ER to annual sums can substantially affect the annual NEE. We estimated that using the daytime temperature response parameters for estimating nighttime ER in the manual chamber approach would result in an overestimation of the net CO2 uptake (NEE) during the snow-free season by 19% at our study site (Table R3). We have incorporated this estimate into the revised manuscript (L192-196) and included Table R3 as part of the Supplementary material (Supplementary Table 1). Similarly, process-based ecosystem models and remote sensing products commonly also arrive at annual NEE budgets by modeling the separate**

9

**Järveoja et al. Authors’ response to reviewers’ comments**

**component fluxes, i.e. ER and GPP. For that purpose, process-based models as well as remote sensing products rely on modeled ER estimates from field studies (EC and manual chambers) for calibration and validation. Furthermore, while the bias on the annual scale is obviously of great interest, our results indicate even more severe mismatches in measured and extrapolated ER patterns at shorter (hourly to seasonal) time scales which hampers our detailed understanding of short-term responses of ER to external perturbations. Thus, the implications from a bias in ER estimates could be tremendous and largely affect global-scale studies of peatland C cycling. We have adjusted the relevant Discussion in the revised version of our manuscript (L186ff).**

**Table R3. Cumulative sums of ecosystem respiration (ER), gross primary production (GPP) and net CO2 exchange (NEE) for the key phenophases and the snow-free season averaged over 2015-2017. In the Model estimate, hourly ER is simulated using the daytime parameters (during both day- and nighttime) from the Lloyd & Taylor (1994) respiration model (see equation in Methods) built on the relationships between measured autochamber ER and air temperature (see parameter values in Fig. 3 of the manuscript). In the AC estimate, ER is calculated based on the hourly measured data (during both day- and nighttime) from the dark autochambers. GPP is derived from the difference between the modeled (in Model) or measured (in AC) daytime ER and the daytime NEE measured concurrently with transparent autochambers. NEE is obtained from the sum of measured daytime NEE and either modeled (Model) or measured (AC) nighttime ER (which equals NEE in the absence of photosynthesis). Note that the aim of this analysis was to investigate the potential bias from extrapolating manual daytime dark chamber measurements to the diel and seasonal scales using constant ER-Ta relationships during both day- and nighttime. We therefore assume that manual chamber measurements were conducted during each daytime hour in order to exclude additional confounding effects due to the relatively lower sampling frequency inherent to the manual chamber technique.**

**Snow-free**

**Green-up	Peak season	Senescence	Dormancy	season**

**Model AC	Model AC	Model AC	Model AC	Model AC**

**ER**	12.6	14.3	16.0	17.8	14.7	18.7	2.7	4.4	46.0	55.2

**GPP** -29.0 -28.9 -37.2 -37.2 -33.0 -33.2 -3.1 -3.1 -102.4 -102.5

**NEE**	-16.4	-14.6	-21.2	-19.4	-18.3	-14.5	-0.4	1.3	-56.4	-47.3

**NEEdiff 1.8 g (12%)	1.8 g (9%)	3.8 g (26%)	1.7 g (131%)	9.1 g (19%)**

**Action: We have included an estimate of bias in the net CO2 sink from assuming a uniform temperature response in the manual chamber approach (Table R3) in the Supplementary material (Supplementary Table 1) and extended the Discussion section on the potential ER bias impact on the mire C balance in the revised manuscript version (L186ff).**

L182-185: However, wasn’t the magnitude of Ra larger than that of Rh (i.e., Figure 4)? Unless I’m not interpreting correctly, this sentence seems to highlight the magnitude of Rh over Ra.

**Response: This statement refers to a comparison of mire Ra and Rh relative to Ra and Rh in other ecosystems, not to a comparison between Ra and Rh within mires. We have revised this and the following sentence for clarity (L242-245).**

10

Järveoja et al. Authors’ response to reviewers’ comments

L244: By “recent root exudates”, do you mean exudates that were released during trenching? The trenching was done almost 2 years before measurements started, but was there any evidence of a disturbance effect from the trenching on respiration?

**Response: ‘Recent root exudates’ refers to assimilates from photosynthesis allocated and released belowground and serving as substrate supply to microbial microorganisms under natural settings. Since vegetation was, however, absent within the Rh plots, the fraction of Rh originating from this substrate supply via plant root exudates was not accounted for in our Rh estimates.**

**The system was operating already in 2014 for a short period. A comparison of Rh fluxes during 2014-2016 suggests that the initial clipping in 2013 had no noticeable effect on Rh in the subsequent years.**

**We have clarified these points in the revised manuscript (1311-316).**

L250: Suggest providing the area of the chamber earlier in the Methods (i.e., L227).

**Response: We followed the reviewer suggestion and now present this information earlier in the Methods section (1299).**

L257: Check spelling on “chamber”.

**Response: Thank you, we have corrected the spelling (1330).**

L268: Is this climate station in the mire itself or elsewhere?

**Response: Yes, the climate station is located in the mire itself. We have clarified this in the revised manuscript: ‘available from a nearby (~50 m) climate station’ within the mire (1342).**

L267-268: I believe that relationships between Rh and Ra vs soil temperature were only investigated for a few of these depths (10, 18, 26 cm). What were the temperature data measured at the other depths used for?

**Response: The six measurement depths (i.e. 2, 10, 18, 26, 34 and 42 cm) are routinely measured at the site within other ongoing research projects. We did initially consider all 6 levels in our analysis, however, chose to present only results from the 10-26 cm depths which turned out to be the most relevant in explaining our fluxes (i.e. Rh). Specifically, soil temperature at the 2 cm depth was highly correlated to Ta while soil temperatures below 26 cm showed no diel variation. These depths were thus not considered further. We have clarified this in the revised manuscript (1414­416).**

L275-276: Were the measurements across the 12 chambers taken at random over the hour, or were they sequential (i.e., all the clear chambers measured first, then all the dark chambers, then dark trenched chambers)?

**Response: The chambers were measured in a fixed sequence which within each of the 4 replicate groups was as follows: first a clear chamber NEE measurement, followed by the adjacent dark chamber ER measurement and concluded by the adjacent dark chamber Rh measurement. The**

11

Järveoja et al. Authors’ response to reviewers’ comments

**sequence of cycling through the groups (group 1 to group 4) was also kept constant. We have clarified this in the revised manuscript version (L333-334).**

L490: What does the standard error represent? Variation across years? Across replicate chambers?

**Response: The standard error represents the variation for a given hour resulting from the variation within each phenophase and across the 3 years. We have clarified this in all relevant figure captions (i.e. Figs. 1 and 4, Supplementary Figs. 1, 2 and 6).**

L513-524: What do the colors (blue to yellow) represent?

**Response: These colors represent the coherence (i.e. the strength of the relationship) between the flux and its environmental control. More specifically, yellow = strong and blue = absent. We have clarified this by including a colormap legend in all relevant figures (Fig. 5 and Supplementary Figs. 7 and 8).**

Figure 1 – It would be helpful to indicate when day vs night is on these figures as it changes over the season (i.e., include grey shading over nighttime hours).

**Response: Following the reviewer’s suggestion, we have added a dark shade over the nighttime hours in Figs. 1 and 4.**

12

Järveoja et al. Authors’ response to reviewers’ comments

**REVIEWER 3:**

The authors present claims on a diel pattern in the temperature response of soil and ecosystem respiration on a nothern undrained peatland based on a semi-long-term measurement campaign. The manuscript is well-written, to the point and concise. The authors base their claims on patterns evident in the measured data. If valid, the results will significantly improve estimates of ecosystem respiration on peatlands and thus aid in the effort to predict the present and future carbon balance of peatlands.

However, the authors do not present convincing evidence that the observed pattern (ie. higher base rate and lower temperature response of respiration during the night vs. during the day) is not an artefact caused by accumulation of CO2 into the soil surface during night, which has been observed to cause erroneously high CO2 emission estimates on peatlands in other studies. My recommendation would be to recognize the time periods when this has occurred during the campaign, remove the measurements affected from the analysis and see if the diel pattern still exists.

**Response: We acknowledge and share the reviewers concern regarding the potential problems with chamber measurements during stable nights with large accumulation of CO2 above the mire surface. We have therefore put extensive efforts into quality checking and scrutinizing our data for potential artifacts already prior to the original manuscript submission.**

**To clarify, there are two issues related to nighttime measurements: 1) the accumulation of CO2 resulting in a weakening of the soil-atmosphere concentration gradient and 2) the disturbance of the CO2 concentration profile from the movement of the chamber lid. In the first case, a weaker soil-atmosphere gradient results in reduced respiration fluxes and thus would not explain our nighttime peak in measured fluxes. In the second issue, however, to which we believe the reviewer is referring to, the closing chamber lid disturbs and mixes up the atmospheric layering thereby diluting the higher near-surface concentrations which artificially increases the soil-atmosphere gradient and emission flux (see e.g. Lai et al. 2012; Brændholt et al. 2017). One way to detect this disturbance is by comparing the concentrations before and after the lid closure at a given measurement height. Since our sample tube inlet is fixed at 10 cm above the mire surface, we are able to identify and quantify such disturbance events. Furthermore, we found in our previous study (Järveoja et al. 2018) that this disturbance effect scales linearly with the bias in the flux rate which allows for correcting the fluxes (up to a certain limit of disturbance magnitude beyond which the flux measurement is discarded). We applied this combined correction and filtering approach on the nighttime data and are therefore confident that the observed nighttime flux peak is not an artifact caused by a disturbance effect.**

**However, to follow up on the reviewer’s request and alleviate the related concerns, we created an alternative dataset in which we removed all nighttime data obtained during stable conditions with accumulation of CO2 above the surface. Specifically, we have discarded all fluxes when the ambient nighttime CO2 concentration exceeded 415 ppm (see Fig. R6 below).**

13

**Järveoja et al. Authors’ response to reviewers’ comments**

**Fig. R6. Hourly time series of ambient CO2 concentration showing original data (red) and filtered
data (blue) in which concentrations > 415pm were removed for discarding fluxes during stable
nights with a large accumulation of CO2 above the mire surface in the years 2015-2017.**

**We then replotted the diel fluxes from Fig. 1 of the main manuscript using both the original (i.e.
presented in the manuscript) and filtered data. The results suggest that the same diel pattern
occurs in the filtered data with essentially no difference compared to the original fluxes (Fig. R7).**

**Fig. R7. Diel patterns for ecosystem respiration (ER) measured by autochambers during the key
phenophases of *a* green-up, *b* peak season, *c* senescence and *d* dormancy (spring and autumn)
shown as a mean of the years 2015-2017 before and after filtering with an ambient CO2
concentration threshold of 415 ppm to eliminate nighttime data during stable atmospheric
conditions (ERAC and ERAC filt., respectively). Shaded bands indicate ±1 standard error for a given
hour resulting from the variation within each phenophase and across the 3 years.**

**The small differences between nighttime fluxes in original and filtered data likely result from the
fact that different populations are being compared here, i.e. the respective means are not based**

14

**Järveoja et al. Authors’ response to reviewers’ comments**

**on the same nights and hence the underlying environmental conditions may vary slightly between the two datasets.**

**In addition, when inspecting the diel methane (CH4) fluxes measured concurrently with the same system, one should expect a similar nighttime increase if such methodological artifact should systematically affect our automated nighttime chamber measurements. However, while the nighttime increase in ER is up to 2-fold, such increase is not apparent in the CH4 fluxes (Fig. R8) (after adopting the nighttime correction by Järveoja et al. 2018) which further confirms that our nighttime flux data is not biased by near-surface accumulation during stable conditions.**

**Fig. R8. Mean diel patterns for the methane (CH4) flux measured by autochambers during the key
phenophases of *a* green-up, *b* peak season, *c* senescence and *d* dormancy (spring and autumn)
shown as a mean of the years 2015-2017. Shaded bands indicate ±1 standard error for a given
hour resulting from the variation within each phenophase and across the 3 years.**

**We therefore conclude that the removal of flux data during nights in which strong accumulation of CO2 occurs does not affect our results on the diel patterns of ER, Rh or Ra. Thus, we are convinced that the data and results presented in this manuscript are not biased by artifacts in relation to nighttime atmospheric conditions.**

**Cited reference: Lai et al. (2012) The effect of atmospheric turbulence and chamber deployment period on autochamber CO2 and CH4 flux measurements in an ombrotrophic peatland. Biogeosciences 9:3305–3322.** [**https://doi.org/10.5194/bg-9-3305-2012**](https://doi.org/10.5194/bg-9-3305-2012)

**Brændholt et al. (2017) Overestimation of closed-chamber soil CO2 effluxes at low atmospheric turbulence. Biogeosciences 14:1603–1616,** [**https://doi.org/10.5194/bg-14-1603-2017**](https://doi.org/10.5194/bg-14-1603-2017)

**Järveoja et al. (2018) Partitioning of the net CO2 exchange using an automated chamber system reveals plant phenology as key control of production and respiration fluxes in a boreal peatland. Global Change Biology 24:3436–3451,** [**https://doi.org/10.1111/gcb.14292**](https://doi.org/10.1111/gcb.14292)

I am also not convinced by the authors' reasoning as to the mechanistic cause of the high night-time respiration and would like to see more discussion on the importance of permanently inundated peat layers to the total ecosystem respiration.

**Response: We interpret this remark as a general comment that is summarizing the specific comments below. We therefore address these concerns in our detailed responses to the specific**

15

**Järveoja et al. Authors’ response to reviewers’ comments**

**points raised below. In brief, we i) provided evidence above (Figs. R6-8) confirming that our measured nighttime fluxes are not the result of artificial bias due to CO2 accumulation, ii) clarify the role of soil temperature as a driver of Rh (L228-242) and iii) extend our discussion on the importance of the contributions from inundated peat layers to ER (L233ff).**

Specific comments follow:

L56-57 This claim would benefit from a couple of references

**Response: We have incorporated the references of Lassplop et al. (2012) and Wohlfahrt & Galvagno (2017) to support this statement (L59).**

L99-101 The Lloyd-Taylor paper discussing exponential models of ER is referenced in this manuscript; why is the improved exponential respiration model presented in that paper used here?

**Response: Thank you for bringing this to our attention. Following the reviewer’s suggestion we now use the improved exponential respiration model from Lloyd & Taylor (1994) to describe the response of ER, Rh and Ra to air or soil temperature in the revised manuscript (L380ff, Figs. 3 and 6, Supplementary Figs. 5 and 12).**

L111-113 How sure are you that the night-time peak is not due to accumulation of CO2 in the surface layer during still clear nights during the summer?

**Response: As described and illustrated above, this nighttime peak was observed even after removing flux data during nights in which strong accumulation of CO2 occurred (Fig. R7). Meanwhile, as we show above in Fig. R8, such nighttime peak is absent in the diel CH4 fluxes. Thus, we are confident that the nighttime peak in our respiration estimates is not the result of a systematic artifact in relation to nighttime atmospheric conditions and near-surface accumulation of CO2.**

L121-125 This relatively weak correlation leaves me unconvinced that such a large effect on Rh could be caused by the rather small variations in soil temperatures. I would like to see the measured temperatures in a plot of some kind of deviation from the daily mean per layer and how those deviations occur over the course of the day, on average over the measurement campaign.

**Response: It is important to recognize that our Rh measurements at the soil surface represent the bulk of various decomposition rates and dynamics occurring simultaneously at various peat depths. For that reason, it is unlikely that a temperature at one single depth could explain well the bulk Rh flux integrating all these decomposition dynamics across the entire profile. More specifically, while our correlations of bulk Rh to temperature at a given depth are averaged across the entire diel scale, in reality, different peat layers might dominate the contributions to bulk Rh at various times within the day. For instance, bulk Rh is likely dominated by Rh in the upper near-surface layer (e.g. 0-5 cm) during the daytime when surface layers warm up, while during nighttime, the contribution is higher from Rh from deeper layers where temperature peaks during evening/night. Thus, given the alternating contributions from decomposition in different peat layers to bulk Rh, the low correlation between total Rh and temperature at a given depth observed in our study is little surprising. For comparison, potentially higher relationships could be expected between diel temperature and decomposition rates occurring within a given peat layer**

16

**Järveoja et al. Authors’ response to reviewers’ comments**

**(as often reported from experimental laboratory studies). In addition, for the same reason one must recognize that while small diel variations of peat temperature result in small diel variation in decomposition rates at a given depth, our bulk Rh measurement from the mire surface represents the integrated sum of all the small diel changes at various depths across the entire peat profile. Thus, the diel variation in bulk Rh is larger than what can be explained by the variation of Ts in one single layer.**

**To follow up on the reviewer’s request we produced a figure (see Fig. R9 below) on the diel temperature deviation from the daily mean for the 2, 10, 18 and 26 cm peat layers averaged over the measurement campaign.**

**Action: In the Discussion section of the revised manuscript we now further stress the limitations and complexity in relating surface Rh to peat temperature measured at a single depth as a driver and highlight that our bulk Rh measurements from the mire surface represent the integral of various decomposition processes related to different temperature dynamics across the peat profile (L228-242). We also added text discussing the contribution of Rh from saturated peat layers to total ER (L233ff).**

**Fig. R9. Mean diel variation in soil temperature (Ts) deviation from the diel mean at 2, 10, 18 and 26 cm depths shown as a mean over the entire snow-free season in 2015 and 2016.**

L175-177 The peat layers below 13 cm are permanently inundated in this bog, according to Fig. 1 in Järveoja et al. 2018. How can the temperature in these layers affect ER as measured in CO2 emission in this magnitude (10 mg C m-2 h-1, re Fig. 1 in this manuscript)?

**Response: We would like to first clarify that this specific sentence discussed the temperature response of Rh rather than that of ER. Furthermore, here we refer not to soil temperature (Ts) at one certain depth but instead to Ts dynamics at any depth above or below the mean water table level. We argue that bulk peat temperature follows air temperature with some temporal lag which overall results in warmer Ts and subsequently higher Rh during the night, rather than during the day. Since the reviewer refers to ER, we note that ER is the sum of Ra and Rh. As seen in Fig. 4 in the main manuscript, nighttime Ra contributes about 50% to nighttime ER.**

17

**Järveoja et al. Authors’ response to reviewers’ comments**

**With regards to the temperature response of Rh, we would like to clarify that out of the available levels (2, 10, 18, 26, 36 and 42 cm) the highest Welch’s cross power spectral density values were in fact observed with Ts at 10 cm depth. We caution that these results do not imply that Ts at this depth should be considered as the main and only driver of bulk Rh nor that most of CO2 is produced at this depth. Instead, the temperature dynamics at this specific depth might merely coincide best with the mean of all diel Rh patterns across the entire peat profile.**

**Furthermore, we would like to note that contrary to the common belief, CO2 production in water-saturated peat is usually not negligible and may be in the order of 10-50% of that obtained with O2 as e-acceptor (Moore & Dalva 1997; Waddington et al. 2001; Blodau et al. 2004; Glatzel et al. 2004; Nilsson & Öquist 2009; Hoyos-Santillan et al. 2016). Furthermore, significant mineralization rates might persist into rather deep layers of the peat profile (e.g. up to ~1 m) and thus, the integrated contribution of CO2 from saturated peat layers to total Rh (and ER) could be**

**substantial.**

**In the light of this complexity, our main intention is not to identify one depth as main control on Rh but to stress that i) the bulk Rh from the surface represents the integral of decomposition dynamics across the peat profile and ii) that the bulk peat temperature lags behind that of air temperature resulting in warmer conditions and elevated Rh during evening/nighttime hours. We improved this discussion in the revised manuscript (L228-242).**

**Action: We have added extra text into the Discussion section for further clarifications of our view on this matter (L228-242).**

**Cited references: Moore & Dalva (1997) Methane and carbon dioxide exchange potentials of peat soils in aerobic and anaerobic laboratory incubations. Soil Biology and Biochemistry 29:1157– 1164.** [**https://doi.org/10.1016/S0038-0717(97)00037-0**](https://doi.org/10.1016/S0038-0717(97)00037-0)

**Waddington et al. (2001) Peat CO2 production in a natural and cutover peatland: Implications for restoration. Biogeochemistry 54:115–130.** [**https://doi.org/10.1023/A:1010617207537**](https://doi.org/10.1023/A:1010617207537)

**Blodau et al. (2004). Carbon turnover in peatland mesocosms exposed to different water table levels. Biogeochemistry 67:331–351.** [**https://doi.org/10.1023/B:BIOG.0000015788.30164.e2**](https://doi.org/10.1023/B:BIOG.0000015788.30164.e2)

**Glatzel et al. (2004) Carbon dioxide and methane production potentials of peats from natural, harvested and restored sites, eastern Québec, Canada. Wetlands 24:261–267.** [**https://doi.org/10.1672/0277-5212(2004)024[0261:CDAMPP]2.0.CO;2**](https://doi.org/10.1672/0277-5212(2004)024%5b0261:CDAMPP%5d2.0.CO;2)

**Nilsson & Öquist (2009) Partitioning Litter Mass Loss into Carbon Dioxide and Methane in Peatland Ecosystems: in Carbon Cycling in Northern Peatlands 184:131–144.** [**https://doi.org/10.1029/2008GM000819**](https://doi.org/10.1029/2008GM000819)

**Hoyos-Santillan et al. (2016) Quality not quantity: Organic matter composition controls of CO2 and CH4 fluxes in neotropical peat profiles. Soil Biology and Biochemistry 103:86–96.** [**https://doi.org/10.1016/j.soilbio.2016.08.017**](https://doi.org/10.1016/j.soilbio.2016.08.017)

18

**Järveoja et al. Authors’ response to reviewers’ comments**

L256-257 This measurement period is very, very short! It makes the measurements sensitive to initial disturbances to the soil surface caused by the closing of the chamber and changing turbulence conditions.

**Response: A three-minute chamber closure is in our view sufficient to capture the linear change in concentrations when measured at 1 Hz frequency. Similar closure periods have been used regularly elsewhere by other studies using chamber systems (e.g. Ojanen et al., 2010; Pumpanen et al., 2015; Görres et al., 2016). However, we are aware of the potential risks for initial disturbance during chamber closure as pointed out by the reviewer and therefore routinely discard the initial 20 seconds after chamber closure to avoid disturbance effects (which we now also clarify in the revised Methods section, L348-349). In addition, it is noteworthy that wind and turbulence effects observed in porous and aerated peat soils such as bogs or drained peatland forests (see Lai et al. 2012; Koskinen et al. 2014) are limited at our wet fen site where the mean water table level is -4.7 cm. During the past years, we have put extensive effort into carefully inspecting our concentration data and we do not observe notable wind disturbance or changes in the concentration build-up after 1 or 2 minutes which would indicate a need for prolonging the closure times (see Fig. R10 as an example of concentration build-up time series). In contrast, there are other arguments that generally promote shorter over longer closure times. This includes changes in headspace temperature and vapor pressure deficit during closure as well as saturation of headspace concentrations which reduces the soil-atmosphere gradient and efflux rates. Thus, we are convinced that our measurement period combined with a thorough quality control is appropriate to obtain reliable flux estimates at our wet fen system.**

**Fig. R10. CO2 concentration time series showing the concentration change during the 3 min
chamber closure period. Each data point represents a 10 sec average of 1 Hz measurements. The
sequence of the chambers is as follows across the 4 groups: clear (NEE), dark (ER) and dark (Rh).**

**Cited references: Ojanen et al. (2010) Soil–atmosphere CO2, CH4 and N2O fluxes in boreal forestry-**
**drained peatlands. Forest Ecology and Management 260:411–421.**
[**https://doi.org/10.1016/j.foreco.2010.04.036**](https://doi.org/10.1016/j.foreco.2010.04.036)

19

**Järveoja et al. Authors’ response to reviewers’ comments**

**Pumpanen et al. (2015) Seasonal dynamics of autotrophic respiration in boreal forest soil estimated by continuous chamber measurements. Boreal Environment Research 20:637–650.**

**Görres et al. (2016) Automation of soil flux chamber measurements: potentials and pitfalls. Biogeosciences 13:1949–1966.** [**https://doi.org/10.5194/bg-13-1949-2016**](https://doi.org/10.5194/bg-13-1949-2016)

**Lai et al. (2012) The effect of atmospheric turbulence and chamber deployment period on autochamber CO2 and CH4 flux measurements in an ombrotrophic peatland. Biogeosciences 9:3305–3322.** [**https://doi.org/10.5194/bg-9-3305-2012**](https://doi.org/10.5194/bg-9-3305-2012)

**Koskinen et al. (2014) Measurements of CO2 exchange with an automated chamber system throughout the year: challenges in measuring night-time respiration on porous peat soil. Biogeosciences 11:347–363.** [**https://doi.org/10.5194/bg-11-347-2014**](https://doi.org/10.5194/bg-11-347-2014)

L287-290 I'm not entirely convinced by the procedure presented in Järveoja et al. 2018, in that the correction term seems very uncertain related to the data points presented. I'd like to see what effect removing the measurements where CO2 accumulation on the surface layer is an issue would have on the night-time Rh and Ra temperature dependency.

**Response: Following the reviewer’s request, we have created an alternative dataset in which we removed all nighttime data during periods with high CO2 accumulation and re-estimated the Rh and Ra temperature dependencies (see Figs. R11 and R12 below). The figures below show that the temperature dependencies remain similar between original and filtered datasets, with marginal differences due to different underlying data populations. This demonstrates that, even if not always accurate for a given single flux estimate, the correction procedure developed in Järveoja et al. (2018) overall effectively removes bias from the mean nighttime fluxes due to CO2 accumulation during stable conditions. Most importantly, this confirms that the nighttime Rh and Ra temperature dependencies presented in our study are not the result of a bias due to CO2 accumulation above the mire during stable nights.**

**Fig. R11. *a-d* (top row) original dataset, *e-h* (bottom row) filtered dataset. Exponential regression
relationships between heterotrophic respiration (Rh) and soil temperature (Ts) at the 10 cm depth**

20

Järveoja et al. Authors’ response to reviewers’ comments

**for day- and nighttime (i.e. photosynthetic photon flux density ≥ 20 and < 20 μmols m^-2^ s^-1^,
respectively). Values shown in the panels (blue and grey for day- and nighttime periods,
respectively) represent model parameters R10 and E0 with standard errors in brackets from the
Lloyd and Taylor (1994) respiration function**. **Solid lines indicate the exponential fit and shaded
bands indicate the 95% confidence intervals.**

**Fig. R12. *a-d* (top row) original dataset, *e-h* (bottom row) filtered dataset. Exponential regression
relationships between autotrophic respiration (Ra) and air temperature (Ta) for day- and
nighttime (i.e. photosynthetic photon flux density ≥ 20 and < 20 μmols m^-2^ s^-1^, respectively). Values
shown in the panels (blue and grey for day- and nighttime periods, respectively) represent model
parameters R10 and E0 with standard errors in brackets from the Lloyd and Taylor (1994)
respiration function. Solid lines indicate the exponential fit and shaded bands indicate the 95%
confidence intervals.**

**Cited reference: Järveoja et al. (2018) Partitioning of the net CO2 exchange using an automated chamber system reveals plant phenology as key control of production and respiration fluxes in a boreal peatland. Global Change Biology 24:3436–3451,** [**https://doi.org/10.1111/gcb.14292**](https://doi.org/10.1111/gcb.14292)

21

**Reviewer comments, second version:**

Reviewer #1 (Remarks to the Author):

Dear authors,

I appreciate the clear and thoughtful response you provided to my initial comments and the ones from the other reviewers. I find these responses - and the changes made to the text - satisfactory. I now recommend this manuscript for publication.


Reviewer #2 (Remarks to the Author):

The authors did an excellent job incorporating the reviewers’ comments and provided very thorough and thoughtful responses to each comment. I have reviewed the revised manuscript and supplemental files and have no additional comments.


Reviewer #3 (Remarks to the Author):

The authors have addressed the issues raised in the first peer review-round well, and I see no reason not to publish the paper.

**Author rebuttal, second version:**

**We sincerely thank the three reviewers for their positive comments. We are glad to hear that all of their earlier concerns have been addressed to their satisfaction in our revised manuscript.**

**Reviewer comments, third version:**

**Author rebuttal, third version:**
